# Remote Sensing of Droplet Number Concentration in Warm Clouds: A Review of the Current State of Knowledge and Perspectives

**DOI:** 10.1029/2017RG000593

**Published:** 2018-06-27

**Authors:** Daniel P. Grosvenor, Odran Sourdeval, Paquita Zuidema, Andrew Ackerman, Mikhail D. Alexandrov, Ralf Bennartz, Reinout Boers, Brian Cairns, J. Christine Chiu, Matthew Christensen, Hartwig Deneke, Michael Diamond, Graham Feingold, Ann Fridlind, Anja Hünerbein, Christine Knist, Pavlos Kollias, Alexander Marshak, Daniel McCoy, Daniel Merk, David Painemal, John Rausch, Daniel Rosenfeld, Herman Russchenberg, Patric Seifert, Kenneth Sinclair, Philip Stier, Bastiaan van Diedenhoven, Manfred Wendisch, Frank Werner, Robert Wood, Zhibo Zhang, Johannes Quaas

**Affiliations:** ^1^ School of Earth and Environment University of Leeds Leeds UK; ^2^ Leipzig Institute for Meteorology Universität Leipzig Leipzig Germany; ^3^ Department of Atmospheric Sciences Rosenstiel School of Marine and Atmospheric Science Miami FL USA; ^4^ NASA Goddard Institute for Space Studies New York NY USA; ^5^ Department of Applied Physics and Applied Mathematics Columbia University New York NY USA; ^6^ Department of Earth and Environmental Sciences Vanderbilt University Nashville TN USA; ^7^ Space Science and Engineering Center University of Wisconsin‐Madison Madison WI USA; ^8^ Royal Netherlands Meteorological Institute De Bilt The Netherlands; ^9^ Department of Atmospheric Science Colorado State University Fort Collins CO USA; ^10^ Rutherford Appleton Laboratory Harwell UK; ^11^ Department of Physics University of Oxford Oxford UK; ^12^ Leibniz Institute for Tropospheric Research Leipzig Germany; ^13^ Department of Atmospheric Sciences University of Washington Seattle WA USA; ^14^ Chemical Sciences Division, Earth System Research Laboratory National Oceanic and Atmospheric Administration Boulder CO USA; ^15^ Deutscher Wetterdienst Lindenberg Germany; ^16^ School of Marine and Atmospheric Sciences Stony Brook University Stony Brook NY USA; ^17^ NASA Goddard Space Flight Center Greenbelt MD USA; ^18^ NASA Langley Research Center Hampton VA USA; ^19^ Institute of Earth Sciences The Hebrew University of Jerusalem Jerusalem Israel; ^20^ Department of Geoscience and Remote Sensing Delft University of Technology Delft The Netherlands; ^21^ Department of Earth and Environmental Engineering Columbia University New York NY USA; ^22^ Center for Climate Systems Research Columbia University New York NY USA; ^23^ Joint Center for Earth Systems Technology Baltimore MD USA; ^24^ Physics Department UMBC Baltimore MD USA

**Keywords:** cloud droplet concentrations, satellite, radar, lidar, remote sensing, passive retrievals

## Abstract

The cloud droplet number concentration (N_d_) is of central interest to improve the understanding of cloud physics and for quantifying the effective radiative forcing by aerosol‐cloud interactions. Current standard satellite retrievals do not operationally provide N_d_, but it can be inferred from retrievals of cloud optical depth (τ_c_) cloud droplet effective radius (r_e_) and cloud top temperature. This review summarizes issues with this approach and quantifies uncertainties. A total relative uncertainty of 78% is inferred for pixel‐level retrievals for relatively homogeneous, optically thick and unobscured stratiform clouds with favorable viewing geometry. The uncertainty is even greater if these conditions are not met. For averages over 1° ×1° regions the uncertainty is reduced to 54% assuming random errors for instrument uncertainties. In contrast, the few evaluation studies against reference in situ observations suggest much better accuracy with little variability in the bias. More such studies are required for a better error characterization. N_d_ uncertainty is dominated by errors in r_e_, and therefore, improvements in r_e_ retrievals would greatly improve the quality of the N_d_ retrievals. Recommendations are made for how this might be achieved. Some existing N_d_ data sets are compared and discussed, and best practices for the use of N_d_ data from current passive instruments (e.g., filtering criteria) are recommended. Emerging alternative N_d_ estimates are also considered. First, new ideas to use additional information from existing and upcoming spaceborne instruments are discussed, and second, approaches using high‐quality ground‐based observations are examined.

## Key Points

- Satellite cloud droplet concentration uncertainties of 78% for pixel‐level retrievals and 54% for 1 by 1 degree retrievals are estimated
- The effective radius retrieval is the most important aspect for improvement, and more in situ evaluation is needed
- Potential improvements using passive and active satellite, and ground‐based instruments are discussed

## 1 Introduction

Clouds are of central importance to the Earth's energy budget. To first order, they are described by fractional coverage, and the zeroth and third moments of the particle size distribution, that is, particle number concentration and water content. In practice, warm (liquid water) clouds are characterized using the vertical integral of the liquid water content (*L*, often expressed in g/m^3^), which is known as the liquid water path (LWP), and the cloud droplet number concentration (*N*_d_, usually in cm^−3^). *N*_d_ is a critical indicator of the impact of aerosol particles (hereafter referred to as aerosols) on cloud microphysical and optical properties. For a given distribution of the dynamical forcing (updraft, *w*), changes in *N*_d_, driven by changes in both aerosol particle number and physicochemical properties, change cloud albedo via the Twomey (1974) effect. *N*_d_ changes also impact cloud macrophysical properties in numerous ways, most of which are currently poorly understood and inadequately represented in large‐scale models (Boucher et al., 2013; Rosenfeld, Andreae, et al., 2014). A reliable remote sensing retrieval of *N*_d_ from ground, and especially from satellites, would be a major step forward in advancing cloud science questions due to vastly increased spatial and temporal sampling. Even uncertain retrievals would be very useful, in particular if errors are well characterized, given the large uncertainties in atmospheric models.

Warm clouds are thought to be the primary mediator of aerosol radiative forcing via aerosol‐cloud interactions (e.g., Heyn et al., 2017), and most aerosol impacts on such clouds are realized via *N*_d_ changes. Moreover, it has been shown that *N*_d_‐driven macrophysical cloud changes (changes in cloud height, depth, and cover) can result because *N*_d_ is a primary control on the sedimentation of cloud droplets and the formation of precipitation, and both factors impact cloud dynamics. The resulting albedo changes can be of either sign (Ackerman et al., 2004) and are comparable in magnitude to the Twomey (1974) effect according to climate models (Lohmann & Feichter, 2005). Overall solar cloud reflectance perturbations due to anthropogenically driven increases in *N*_d_ are complex and nonlinear, depending not only on the magnitude of the *N*_d_ perturbation but also, for example, on the pristine atmospheric state (Carslaw et al., 2013), the cloud optical depth, *τ*_c_ (Platnick & Twomey, 1994), and the degree to which clouds are precipitating (Chen et al., 2014). Accurate collocated observations of *N*_d_ and macrophysical cloud properties would provide an important resource for quantifying the response of clouds to aerosols and for validating these processes in models. Some progress on this has been made but is hampered by questions regarding observational uncertainties (Gryspeerdt et al., 2016, 2017; Quaas et al., 2006). *N*_d_ is especially useful in this regard since aerosol retrievals from passive instruments are not currently possible in cloudy pixels and are problematic when near to clouds (Christensen et al., 2017; Gryspeerdt et al., 2017; Remer et al., 2005; Twohy et al., 2009; Várnai & Marshak, 2009).

In atmospheric climate models, cloud macrophysical properties are characterized by their fractional coverage and by their liquid‐ and ice water contents (specific mass concentration). Cloud microphysical parameterizations of increasing complexity are being implemented that simulate the cloud particle number concentration and its dependence upon aerosols (Khain et al., 2000). Today, almost all climate models include a representation of aerosol‐cloud interactions (e.g., Ghan et al., 2016; Gryspeerdt et al., 2017; Penner et al., 2006; Quaas et al., 2009), yet there has been little systematic effort to evaluate *N*_d_ in large‐scale models and to constrain it using observations. A particular problem is that climate models often impose a lower bound on *N*_d_ that artificially reduces sensitivity to aerosol perturbations (Hoose et al., 2009). The spread between models in regional mean *N*_d_ can exceed 1 order of magnitude in regions of extensive warm low clouds impacted by regional pollution (Ban‐Weiss et al., 2014; Wyant et al., 2015). There is also a need to evaluate *N*_d_ in regional and higher resolution models; satellite observations of *N*_d_ have proven to be an important resource for this (George et al., 2013; Grosvenor et al., 2017) since other forms of *N*_d_ observations are often not available or are limited.

In warm clouds, *N*_d_ is determined by (i) the activation process occurring (primarily) at cloud base (and therefore by the number concentration, size distribution, and physicochemical properties of aerosol particles, as well as cloud updraft speeds); (ii) evaporation due to lateral and cloud top entrainment and due to warming (e.g., in downdrafts); (iii) losses due to coalescence; and (iv) wet removal via collection by precipitation. At the process level and when thermodynamic equilibrium can be assumed, cloud droplet activation is sufficiently described by Köhler theory. However, there are details still to be worked out for complex internal mixtures of soluble and insoluble aerosols and aerosols with weakly soluble coatings. Since the in‐cloud residence time in warm clouds is typically small (of order of 10^3^ s), losses of *N*_d_ and consequently a reduction of the number concentration of aerosol are limited. However, via Cloud Condensation Nuclei (CCN) loss, coalescence scavenging can have a significant effect on *N*_d_ on daily time scales (10^5^ s; Feingold et al., 1996). Thus, in a warm cloud, *N*_d_ is primarily determined by the activation process and, in laterally entraining clouds such as cumulus, additionally also by evaporative losses due to the entrainment of dry air.

However, important questions remain when modeling droplet activation. This concerns the difficulties of models in properly representing the vertical wind at cloud scale (Donner et al., 2016; Tonttila et al., 2011) and questions about the accuracy of some of the activation schemes used in climate models (e.g., Simpson et al., 2014). Analytical and quasi‐analytical formulations exist that diagnose *N*_d_ as a function of updraft speed, and parameters describing the aerosol particle size distribution and chemical composition (Abdul‐Razzak & Ghan, 2000; Barahona & Nenes, 2007; Fountoukis & Nenes, 2005; Twomey & Squires, 1959). For a given framework, different parameterizations produce similar results (Ghan et al., 2011). However, activation schemes need more testing against observations under real environmental conditions and with observed updrafts, aerosol composition, and size distributions. Some CCN‐*N*_d_ “closure” experiments have been performed that predict *N*_d_ from CCN and updraft measurements and evaluate the prediction with independent measurements of *N*_d_ (e.g., Conant et al., 2006; Snider et al., 2003) showing agreement of the parcel models with in situ observations to within 20% (Fountoukis et al., 2007).

This paper critically reviews the current approaches for satellite *N*_d_ estimates (section 2), highlights progress that has been made in addressing outstanding issues, assesses currently available data sets (section 3), and discusses some promising alternative methods from satellite (section 4) and ground‐based (section 5) remote sensing.

## 2 Retrieval of N_d_ From Passive Satellite Observations

The commonly used method for inferring *N*_d_ from passive satellite observations utilizes retrievals of cloud optical depth, *τ*_c_ and of cloud droplet effective radius (*r*_e_, Nakajima & King, 1990), and cloud top temperature (see sections 4 and 5 for an overview of other methods). The methods built upon the initial work of Brenguier et al. (2000), Han et al. (1998), Nakajima et al. (2001), Schüller et al. (2003, 2005), and Szczodrak et al. (2001) and were subsequently applied to larger *N*_d_ data sets in Bennartz (2007), Boers et al. (2006), Quaas et al. (2006), and most recently Bennartz and Rausch (2017) and a data set based on the methods described in Grosvenor and Wood (2014) and Grosvenor et al. (2018; see section 3 for a comparison of the latter two data sets). The technique relies upon the assumptions that (i) throughout their depth, clouds have liquid water contents (*L*) that are a constant fraction of that expected from adiabatic uplift and that (ii) *N*_d_ remains vertically constant.

Here the simplest retrieval technique is derived; Boers et al. (2006) introduced methods to utilize more complicated profiles (see sections 2.3.4 and 2.3.5). The assumptions required have been shown to hold well for stratocumulus, and the evidence for this will be discussed in more detail later in section 2.3.

### 2.1 Definitions

Here we briefly define some of the quantities used for the *N*_d_ retrieval; for further details we refer to reader to Wendisch and Yang (2012). Units for all quantities in the equations in this paper are SI units.

*τ*_c_ is a unitless quantity that is defined as the vertical integral of the cloud extinction coefficient, *β*_ext_ (in meters^−1^), between cloud base and cloud top, which we denote here as *z*_base_ and *z*_top_ (all in meters), respectively, with cloud geometrical thickness *H* = *z*_top_−*z*_base_:

(1) $$\tau_{c}=\int_{z_{\text{base}}}^{z_{\text{top}}}\beta_{\text{ext}}(z)\;dz.$$

The wavelength dependence is implicit, and cloud optical properties are defined as averages over the solar spectrum. Assuming spherical cloud droplets, *β*_ext_(*z*) can be expressed as

(2) $$\beta_{\text{ext}}(z)=\pi \int_{0}^{\infty }Q_{\text{ext}}(r)\;r^{2}\;n(r)\;dr,$$

where *r* is the droplet radius (in meters) and *n*(*r*) (in m^−4^) is the droplet number size distribution within a cloud unit volume at the height *z*. It is related to the droplet number concentration per unit volume, *N*_*d*_, here in m^−3^, such that

(3) $$N_{d}(z)=\int_{0}^{\infty }\;n(r)\;dr.$$

*Q*_ext_(*r*) is the unitless extinction efficiency factor, which represents the ratio between the extinction and the geometric cross section of a given droplet. The geometric optics limit is almost reached because *r* ≫ *λ*, with *λ* being the wavelength of light concerned (typically centered at 0.65–0.86 μm). Thus, *Q*_ext_ can be approximated by its asymptotic value of 2 (van de Hulst, 1957).

The droplet effective radius *r*_e_(*z*) (Hansen & Travis, 1974) and liquid water content *L*(*z*) at a given height are defined as

(4) $$r_{e}(z)=\frac{\int_{0}^{\infty }r^{3}\;n(r)\;dr}{\int_{0}^{\infty }r^{2}\;n(r)\;dr}$$

and

(5) $$L(z)=\frac{4\pi \;\rho_{w}}{3}\int_{0}^{\infty }r^{3}\;n(r)\;dr,$$

where *ρ*_w_ = 1,000 kg/m^3^ is the density of liquid water and L is in kg/m^3^.

### 2.2 Adiabatic Cloud Model

Combining equations (4) and (5) and inserting into equation (2) gives

(6) $$\beta_{\text{ext}}(z)=\frac{3\;Q_{\text{ext}}}{4\;\rho_{w}}\frac{L(z)}{r_{e}(z)}.$$

The determination of the dependence of *r*_e_(*z*) on *L*(*z*) and *N*_d_(*z*) utilizes the fact that the “*k*” value, which relates the volume‐mean droplet radius *r*_v_ (see below for more detail) to *r*_e_,

(7) $$k={\left(\frac{r_{v}}{r_{e}}\right)}^{3},$$

appears approximately constant in stratocumulus clouds; the validity of this assumption is discussed in more detail in sections 2.3.2 and 2.4.4. One can write *r*_v_ as

(8) $$r_{v}^{3}(z)=\frac{1}{N_{d}(z)}\int_{0}^{\infty }r^{3}\;n(r)\;dr=\frac{3\;L(z)}{4\;\pi \;\rho_{w}\;N_{d}(z)}=k\;r_{e}^{3}(z),$$

where we have used equation (5) to insert *L* and equation (7) to write *r*_v_ as a function of *k* and *r*_e_. The following utilizes the assumption that *N*_d_(*z*) is constant with height (see discussion in section 2.3.5) and that *L*(*z*) is a constant fraction, *f*_ad_, of its adiabatic value. The latter equates to

(9) $$L(z)=f_{\text{ad}}\;c_{w}\;z,$$

where *c*_w_ is the rate of increase of *L* with height (d*L*/d*z*, with units kg/m^4^) for a moist adiabatic ascent and is referred to as the “condensation rate” in Brenguier et al. (2000) or the “water content lapse rate” in Painemal and Zuidema (2011). It is a constant for a given temperature and pressure and is discussed further in sections 2.3.3 and 2.3.4. Allowing these assumptions, using equation (8) to substitute for *r*_e_ in equation (6), and combining with equations (1) and (9), we obtain

(10) $$\begin{array}{ccc} \tau_{c} & =\int_{z_{\text{base}}}^{z_{\text{top}}}Q_{\text{ext}}{\left(\frac{3\;f_{\text{ad}}\;c_{w}}{4\;\rho_{w}}\right)}^{2/3}{\left(N_{d}\;\pi \;k\right)}^{1/3}z^{2/3}dz & \\ & =\frac{3\;Q_{\text{ext}}}{5}{\left(\frac{3\;f_{\text{ad}}\;c_{w}}{4\;\rho_{w}}\right)}^{2/3}{\left(N_{d}\;\pi \;k\right)}^{1/3}H^{5/3}. \end{array}$$

All that remains now is to relate the cloud geometrical depth, *H*, to the *r*_e_ value obtained from satellite. It is assumed that the retrieved *r*_e_ is that at cloud top, that is, *r*_e_(*z*_top_). Platnick (2000) showed that the *r*_e_ obtained by the MODerate Imaging Spectroradiometer (MODIS; Salomonson et al., 1998) and other shortwave infrared (IR)‐based retrievals of *r*_e_ are heavily weighted toward the top of a cloud, although the exact vertical weighting depends on the wavelength of the absorbing shortwave‐IR channel used (the more absorbing the less penetration into the cloud) and on the cloud extinction profile. Section 2.3.1 discusses the error introduced by such issues. Then, we can use equations (8) and (9) applied for cloud top (*z* = *z*_top_) to specify *H* as a function of *r*_e_(*z*_top_), *N*_d_ and other known parameters. Finally, rearranging for *N*_d_ gives

(11) $$\begin{array}{c} N_{d}=\frac{\sqrt{5}}{2\pi \;k}{\left(\frac{f_{\text{ad}}\;c_{w}\;\tau_{c}}{Q_{\text{ext}}\;\rho_{w}\;r_{e}^{5}}\right)}^{1/2}. \end{array}$$

It is worth noting that *r*_e_ is raised to the power of $-\frac{5}{2}$ in this expression, compared to *τ*_c_, *f*_ad_, and *c*_w_ being raised only to a power of $\frac{1}{2}$ and *k* to the power of −1. Thus, *N*_d_ retrieved in this way is very sensitive to *r*_e_ and, therefore, to uncertainties in *r*_e_, although uncertainties in the other variables might also be considerable.

The rest of the subsections in section 2 discuss the various known aspects of the *N*_d_ retrieval uncertainty. These are grouped to firstly assess potential problems with the adiabatic cloud model (section 2.3) and secondly errors related to the retrieval of *τ*_c_ and *r*_e_ (section 2.4). Some validation studies of various *N*_d_ products are discussed in section 2.5, although the number of such studies is currently very limited. Finally, in section 2.6 we present an estimate of the overall uncertainty. This is assessed firstly for individual pixel‐level retrievals from the MODIS instrument (1‐km resolution at nadir), and then we go on to estimate how this changes upon averaging over larger areas (1° ×1°). We restrict the analysis to the “best case scenario” of relatively homogeneous warm stratocumulus clouds in situations where the solar zenith angle (SZA) is low (i.e., the Sun is high in the sky), for viewing angles below 55° and when *τ*_c_> 5. The latter restriction is due to the high degree of uncertainty in *r*_e_ retrievals arising from a high sensitivity of cloud reflectance (used to retrieve *τ*_c_ and *r*_e_) to cloud *τ*_c_ and *r*_e_ (Sourdeval et al., 2016; Zhang & Platnick, 2011) for optically thin clouds, along with increased sensitivity to uncertainties in the surface albedo. The reasons for the former restrictions are explained in the following sections.

### 2.3 N_d_ Errors Related to the Adiabatic Cloud Model

#### 2.3.1 Inconsistencies Between Vertically Stratified Models

A conceptual issue when estimating *N*_d_ from most usual retrievals of *τ*_c_ and *r*_e_ lies in an intrinsic inconsistency between the vertical distribution of cloud properties assumed by the *τ*_c_ and *r*_e_ retrieval and that assumed for the *N*_d_ calculation. The latter assumes a cloud that follows an adiabatic or subadiabatic growth, in which *L* and *r*_e_ monotonically increase toward cloud top. On the other hand, the retrieved *τ*_c_ and *r*_e_ used as inputs to equation (11) are typically retrieved with the assumption that the vertical distribution of *r*_e_ and *L* is homogeneous (King et al., 1998). These two assumptions are incompatible at first glance but can be merged under two conditions. According to the framework described in section 2.1, it is necessary that (i) the retrieved *r*_e_ corresponds to the top of a (sub) adiabatic cloud and (ii) the retrieved *τ*_c_ must still be radiatively representative of the cloud layer when the vertical stratification of the particle size spectrum follows that of the adiabatic model instead of being vertically uniform. The first condition is particularly important due to the strong sensitivity of *N*_d_ estimates on the choice of *r*_e_.

Platnick (2000) showed that the *r*_e_ retrieved by MODIS corresponds to a value that is below cloud top, depending on the penetration depth of the selected shortwave‐infrared channel. They conclude that the *r*_e_ retrievals obtained from the 3.7‐ and 2.1‐μm channels are representative of those at optical depths of about 2 and 3.5 below cloud top, respectively. Grosvenor et al. (2018) calculated such penetration depths (in terms of optical depth) using retrievals performed upon a range of idealized adiabatic clouds and found that they obeyed monotonic functions of the overall cloud *τ*_c_. Using these functions and observed MODIS *N*_d_ data, they estimated the resulting *N*_d_ error globally for 1° ×1° regions and found annual mean *N*_d_ overestimates of around 25–38% for the stratocumulus regions (overall stratocumulus average of 32%) for the 2.1‐μm retrieval with a relative standard deviation in the percentage bias of ∼20–40%. The errors for the 3.7‐μm retrieval were considerably smaller (<20%), although with a higher relative standard deviation of ∼40–60%. It was also predicted that these errors reduce quickly as *τ*_c_ increases, so that the restriction of *N*_d_ retrievals to optically thicker clouds reduces the bias. In this review we assume an *N*_d_ error of 30% for biases due to vertical stratification, but this is likely an overestimate for the 3.7‐μm retrieval.

Retrievals of *r*_e_ and *τ*_c_ can be performed using lookup tables (used to convert the satellite‐observed reflectances into *τ*_c_ and *r*_e_) that are modeled upon adiabatically stratified clouds; this would allow the cloud top *r*_e_ to be returned while taking into account the penetration depth issues (Brenguier et al., 2000; Han et al., 1998; Nakajima et al., 2001; Schüller et al., 2003, 2005; Szczodrak et al., 2001). However, such models are not used operationally as yet. These techniques are discussed further in section 4.1.

#### 2.3.2 The Droplet Spectrum Width (k Parameter)

In the above formulation of equation (11) the *k* parameter links *r*_e_ to the mean volume radius (equation (7)) and, subsequently, to *L* and *N*_d_. For a droplet size distribution (DSD) that follows a modified gamma function,

(12) $$n(r)=N_{0}r^{\frac{1}{v_{e}}-3}\exp\left(-\frac{1}{r_{e}v_{e}}r\right),$$

where N_0_ is a constant and *v*_e_ is the effective variance, the *k* parameter is directly dependent on *v*_e_ such that

(13) $$k={\left(r_{v}/r_{e}\right)}^{3}=(1-v_{e})(1-2v_{e}).$$

Thus, the *k* parameter is a measure of the width of the modified gamma droplet distribution.

For the calculation of *N*_d_ the *k* parameter is assumed constant at least within the area of the pixel and also vertically within the cloud. However, from aircraft observations of stratocumulus in the southeast Pacific, Painemal and Zuidema (2011) found that *k* increased (i.e., a narrowing of the distribution) with height within the clouds toward a value of 0.88 near cloud top, whereas the profile‐averaged value was 0.8. In turn, observations of North Atlantic stratocumulus (Brenguier et al., 2011; Pawlowska et al., 2006) reported droplet spectra that were equally likely to widen as to narrow with height, despite the expectation that droplet spectra growing by water vapor deposition should narrow. Coagulation processes might explain this result. Aircraft studies have also shown some degree of variability of *k* between cloud types with values ranging from 0.67 for continental clouds, 0.80 for marine clouds (Martin et al., 1994; Pawlowska & Brenguier, 2003), and specifically for convective clouds, 0.79 (Freud & Rosenfeld, 2012).

There is also a body of literature that suggests that *k* varies with *N*_d_ or *r*_v_ and a number of parameterizations have been developed, as summarized and compared in Xie et al. (2017). Rotstayn and Liu (2003) and Morrison and Grabowski (2007) parameterize *k* as function of *N*_d_ based upon aircraft data; the former from a variety of campaigns within different cloud regimes (Liu & Daum, 2002) and the latter using the aircraft data from Martin et al. (1994). Liu et al. (2008) give an expression for *k* as function of *r*_v_ based on ground and aircraft data from a variety of locations. In all cases *k* is predicted to decrease with increasing *N*_d_ for a given liquid water content. The range of *k* values as a function of *N*_d_ predicted by the parameterizations (and the data upon which they are based) is large, which would suggest the need for including such effects in satellite *N*_d_ estimates. However, Brenguier et al. (2011) shows that the aircraft observations of the *k* values from the older instruments upon which these studies are based are likely to be biased low and that the bias is likely worse for higher *N*_d_. Thus, the observed relationships are possibly due to instrumental artifacts.

Brenguier et al. (2011) compiled *k* values from multiple studies and found it to be more variable for pristine clouds and more uniform in heavily polluted situations. This implies that there is potentially greater uncertainty in retrieved *N*_d_ due to *k* for pristine cloud scenes. The values of *k* spanned approximately 0.7–0.9, and uncertainties were quantified at 10% to 14%. For stratocumulus, Merk et al. (2016) suggest an upper limit for the uncertainty in *k* of 12%, which is the value that is adopted in this paper.

New capabilities for retrieving *k* from remote sensing using polarimetric measurements are discussed in section 4.3.

#### 2.3.3 Degree of Subadiabaticity and Variable Liquid Water Content Profiles

Although the relative sensitivity of *N*_d_ to errors in *f*_ad_ is low compared to *r*_e_, *f*_ad_ can have significant variability, which increases related uncertainties. Janssen et al. (2011) suggest that *f*_ad_ is among the most significant contributors with an estimate of 25% of the overall *N*_d_ error.

The retrieval of *N*_d_ relies on the assumption that *L* increases linearly with height above cloud base at a constant fraction, *f*_ad_, of that predicted for a moist adiabatic parcel ascent (see equations (9) and (14)). Substantial departures from fully adiabatic profiles (i.e., *f*_ad_=1) have been observed in stratocumulus in many aircraft studies all over the globe (Albrecht et al., 1985; Boers et al., 1998; Brenguier et al., 2000; Ishizaka et al., 1995; Min et al., 2012; Nicholls & Leighton, 1986; Painemal & Zuidema, 2011; Rogers & Telford, 1986); these studies showed that *f*_ad_ varied between 0.1 and 0.9. The magnitude of *f*_ad_ varies with cloud geometrical thickness. For stratocumulus in the southeast Pacific, Min et al. (2012) showed that geometrically thin clouds (<200 m) exhibited a higher mean *f*_ad_ value (∼0.8) than thicker clouds (on the order of 500 m) for which *f*_ad_ decreased to 0.5. Min et al. (2012) attributed this to increased entrainment. To better account for variations in *L* profiles, Boers et al. (2006) developed an ad hoc model that scales *f*_ad_ with cloud geometrical thickness, with $f_{\text{ad}}\to 1$ as H→0.

The advent of routine cloud radar and lidar observations allows for more systematic observations of *f*_ad_. Work by Chin et al. (2000), Kim et al. (2008), Merk et al. (2016), and Politovich et al. (1995) provided systematic values for *f*_ad_ with an average value of *f*_ad_≈0.6. These estimates rely on the retrievals of LWP and *H*. The related retrieval errors result in high uncertainties in individual *f*_ad_ estimates especially for thin clouds (Merk et al., 2016). Averaging over many observations is required to sufficiently improve accuracy.

The two main processes responsible for the departure of liquid water profiles from adiabatic profiles are the mixing of cloudy air with ambient dry air, and the removal of liquid water due to precipitation. Modification of the *L* profile by entrainment at cloud top and cloud sides can also affect the validity of the assumption that the retrieved *r*_e_ is representative of that at the very top of the cloud. Entrainment can result in both homogeneous (both *r*_e_ and *N*_d_ decrease) and inhomogeneous mixing (*L* in the entrainment zone decreases by reduction in *N*_d_ only). Albrecht et al. (2016) investigated cloud top entrainment within stratocumuli by using Doppler cloud radar observations to close the turbulent kinetic energy budget in the entrainment zone. Studies such as this can offer a better estimate of entrainment rates, which improve *L* profile parameterizations but cannot offer insight into the partitioning of the mixing process and its relation to cloud optical parameters.

For the error assessment in this review, the analysis by Merk et al. (2016) is used, who obtained, from ground‐based measurements, a median *f*_ad_=0.66 and a relative standard deviation of 30%.

#### 2.3.4 Condensation Rate

For a parcel ascending under moist‐adiabatic conditions, the condensate rate, *c*_w_, depends on temperature, *T* and pressure, *P* (Ahmad et al., 2013; Albrecht et al., 1990):

(14) $$c_{w}=\rho_{a}\frac{c_{p}}{L_{v}}\left(\Gamma_{m}\left(T,P\right)-\Gamma_{d}\right),$$

where *ρ*_a_ is the parcel air density, $c_{p}=1,004\;J\;{\text{kg}}^{-1}K^{-1}$ is the specific heat of dry air at constant pressure, *L*_v_ is the latent heat of vaporization, and Γ_d_=−*g*/*c*_p_ (*g* = 9.81 m/s^2^ gravitational acceleration) and Γ_*m*_ are the dry and moist temperature lapse rates, respectively. Since *c*_w_ is a weak function of pressure (*P*) and temperature (*T*), it is often assumed constant vertically throughout the cloud and cloud top pressure (*P*_top_) and temperature (*T*_top_) are used to calculate the value of *c*_w_. This assumption is likely to introduce negligible errors. For example, for a 976‐m thick cloud with *τ*_c_=80, *r*_e_=21 μm, *N*_d_=60 cm^−3^, a cloud base pressure of 900 hPa and a cloud base temperature of 283 K, Grosvenor and Wood (2014) calculate an underestimate in *N*_d_ of only 2%, assuming that *c*_w_ is constant throughout the cloud instead of taking into account the temperature and pressure variation. Errors for less deep clouds are even smaller.

*c*_w_ depends more strongly on *T* than on *P*, and therefore, several *N*_d_ retrievals assume a constant *P* value for all clouds given the uncertainties in retrievals of *P*_top_ from passive satellites. For example, King et al. (2013) showed that MODIS‐derived *P*_top_ values consistently overestimated the aircraft observed values for stratocumulus during the VAMOS Ocean‐Cloud‐Atmosphere‐Land Study (VOCALS) campaign by about 250 hPa with no correlation between the two. Thus, the biases introduced by using the retrieved *P*_top_ may be larger than those introduced by assuming a constant *P*_top_. Grosvenor and Wood (2014) showed that the decrease in *N*_d_ associated with a decrease in *P* from 850 to 650 hPa is 8%, 6%, and 4% at temperatures of 283, 273, and 263 K, respectively. This also shows that the pressure dependence is more important for warmer clouds.

In contrast, the decreases in *N*_d_ as temperatures decrease from 283 to 263 K are 24% and 22% at 850 and 650 hPa, respectively (Grosvenor & Wood, 2014). It is important to consider *T*_top_ variation, which can be considerable around the globe, and of larger importance then *P* variations. *T*_top_ retrievals have smaller biases than *P*_top_ retrievals and can more reliably be used in the *N*_d_ calculation.

Still, satellite retrievals of *T*_top_ suffer from errors. King et al. (2013) found that MODIS‐derived *T*_top_ underestimate aircraft observations, with a maximum negative bias of 3.7 K. Min et al. (2012) demonstrated a mean negative MODIS bias of 1.65 K. For overcast scenes Zuidema et al. (2009) found a mean underestimate of 1.3 K for MODIS Collection 4 *T*_top_ retrievals compared to the inversion base temperature from radiosondes. These results span a larger space and time sample than the aircraft results mentioned above, but the result is similar to that from Min et al. (2012).

The above suggests a maximum error in *T*_top_ of 3.7 K, which implies an error in *c*_w_ of 8% at a *T*_top_ and *P*_top_ value of 283 K and 850 hPa, respectively, which we adopt as a representative error for *c*_w_ in this paper.

#### 2.3.5 Assumption of Vertically Constant N_d_

**Figure 1:**
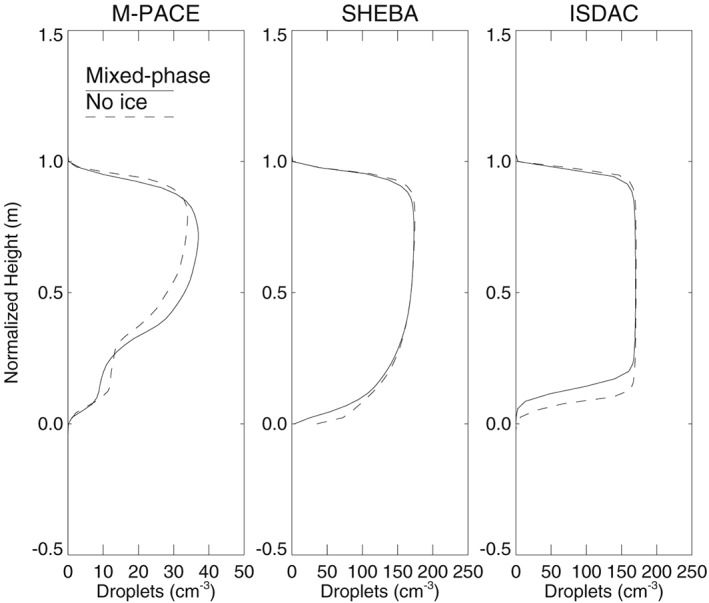
Horizontally averaged N_d_ profiles normalized by cloud base and top heights from the last time step of Distributed Hydrodynamic Aerosol and Radiative Modeling Application (DHARMA) large‐eddy simulation simulations based on the Mixed‐Phase Arctic Cloud Experiment (MPACE; Klein et al., 2009, “standard” and “no ice” cases), the Surface Heat Budget of Arctic Ocean campaign (SHEBA; Morrison et al., 2011, “BASE” and “LOWNI” cases), and the Indirect and Semidirect Aerosol Campaign (ISDAC; Ovchinnikov et al., 2014, “ice4” and “ice0” cases with bulk microphysics).

Observations of vertical cloud structure from aircraft support the approximate validity of the assumption of vertically constant *N*_d_ for stratocumulus (Brenguier et al., 2000; Miles et al., 2000; Painemal & Zuidema, 2011; Wood, 2005). Also large‐eddy simulations (LESs) of stratiform low clouds confirm this statement. Examples are shown in Figure 1, in which the importance of the presence of ice in mixed‐phase clouds for the assumption of vertically constant *N*_d_ is also explored. These simulations demonstrate that although not exactly constant with height, *N*_d_ may commonly be approximately vertically uniform even in the presence of ice.

In contrast to stratocumulus, cumulus may laterally entrain dry air, which leads to increased evaporation of droplets and reduced adiabaticity. Observations of small cumulus (Gerber et al., 2008; Jiang et al., 2008), however, show that droplet concentrations do not decrease with height above cloud base despite subadiabatic liquid water contents. An explanation is that secondary activation above cloud base may help maintain uniform vertical profiles of droplet concentrations in shallow cumuli.

Fields of continental shallow cumuli, with a distribution of cloud top heights, have demonstrated a much wider horizontal variability of *N*_d_ at a fixed elevation than found in stratocumulus, where the cumulus *N*_d_ as a function of height is strongly correlated with *L* in both observations and LESs of observed cases (Endo et al., 2015).

### 2.4 Passive‐Retrieval Errors in τ_c_ and r_e_ and the Impact on N_d_

Errors propagated from passive retrievals of *τ*_c_ and *r*_e_ will generate uncertainties in the subsequently derived *N*_d_. *r*_e_ uncertainties are likely to have a larger impact than *τ*_c_ errors due to the larger sensitivity of *N*_d_ to *r*_e_ that follows from equation (11) (see equation (16)). Retrievals based on MODIS and other instruments employ bispectral algorithms for retrieving *τ*_c_ and *r*_e_ (Nakajima & King, 1990), whereby these quantities are estimated using reflectances from both a nonabsorbing visible wavelength (denoted here as *R*_vis_) and an absorbing shortwave infrared wavelength (*R*_SWIR_). To observe *R*_vis_, the MODIS instrument uses the 0.65‐μm channel over land and the 0.86‐μm channel over the ocean. Three MODIS channels are used for measuring *R*_SWIR_ for these retrievals: 1.6, 2.1 and 3.7 μm. We denote the *r*_e_ retrieved using these different channels as *r*_e1.6_, *r*_e2.1_, and *r*_e3.7_, respectively.

#### 2.4.1 Subpixel Heterogeneity

Retrieval schemes of *τ*_c_ and *r*_e_ from satellite instruments often consist of assuming that each cloud pixel is horizontally homogeneous (e.g., Platnick et al., 2017; Roebeling et al., 2006). However, the horizontal resolution of satellite observations is often much coarser than the spatial variability of the structure and properties of clouds; what is actually measured by satellite instruments corresponds to the average upward radiance reflected by clouds (with contributions from the surface and other atmospheric components) within one satellite pixel. MODIS visible and shortwave‐infrared channels that are used to retrieve *τ*_c_ and *r*_e_ possess a nadir resolution of 250 m (for the 0.65 and 0.86‐μm channels), 500 m (1.6 and 2.1 μm), or 1 km (3.7 μm). The approximation of subpixel homogeneity is known to have substantial retrieval consequences for the visible channel due to the nonlinear relationship between *τ*_c_ and cloud reflectance (e.g., Marshak et al., 2006, hereafter M06), which leads to the so‐called plane‐parallel albedo bias (Cahalan et al., 1994; Kato & Marshak, 2009; Marshak et al., 2006; Oreopoulos & Davies, 1998; Oreopoulos et al., 2007). This results in retrieved *τ*_c_ values that are smaller than the true values. Similar effects on the shortwave‐infrared retrievals lead to an underestimate in *r*_e_ (M06), although the effect is less pronounced because of shortwave absorption at those wavelengths.

**Figure 2:**
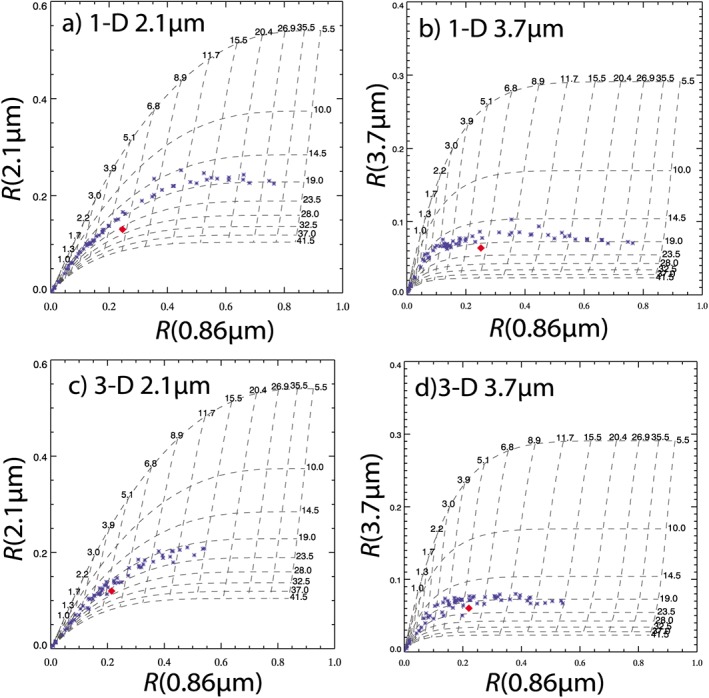
An example of subpixel variability causing overestimates of r_e_ when retrievals are performed at low resolution compared to the true mean value, taken from Zhang et al. (2012). Shown are the 100‐m cloud reflectances (blue asterisks) at a visible wavelength (0.86 μm; x axis; referred to in the text as R_vis_) and a shortwave infrared wavelength (SWIR) wavelength (either 2.1 μm, left column, or 3.7 μm, right column; y axis; R_SWIR_) of a single 800 × 800 m region. The reflectances were generated by applying both a 1‐D (top row) and a 3‐D (bottom row) radiative transfer (RT) model to cloud fields generated by a 100‐m resolution large‐eddy simulation model. The red diamond shows the 800‐m resolution reflectances calculated as the mean of the high‐resolution values. The dotted lines show contours of the τ_c_ (labels running horizontally along the top in each panel) and r_e_ (labels running vertically down the right‐hand sides) that would be retrieved for a given reflectance pair. The values are based on similar calculations to those used for MODerate Imaging Spectroradiometer (MODIS) retrievals and were generated using solar and viewing zenith angles of 20° and 0° (nadir), respectively. It is clear that the high‐resolution r_e_ values are reasonably constant within this region but that there is a large degree of τ_c_ variability. The 1‐D RT r_e_ retrieved at low resolution (around 25 μm for the 2.1 μm retrieval and 20.5 μm for the 3.7 μm one) is significantly higher than the mean of the high‐resolution retrievals (around 19 μm). The 3.7‐μm retrieval is less affected due to the nature of relationships between the reflectances and τ_c_ and r_e_. With 3‐D RT the retrieved low‐resolution r_e_ values are similar to with 1‐D RT, although the high‐resolution values are now higher, which is consistent with the discussion in section 2.4.2.

However, these considerations are strictly only valid if the *τ*_c_ and *r*_e_ retrievals are independent of each other. Yet this is not the case in bispectral retrievals (Nakajima & King, 1990). In this case, diagnosing the effect of subpixel averaging is more complicated with the sign and magnitude of the *r*_e_ and *τ*_c_ errors strongly related to the second partial derivatives of the functional relationships between the retrieved quantities (i.e., *r*_e_ and *τ*_c_) and the reflectances, along with the magnitude of the subpixel variances and covariances of the reflectances (Zhang et al., 2016). The partial derivatives are determined solely by the forward model (the radiative transfer model and cloud assumptions used for the retrieval) and thus do not vary for a given viewing and solar geometry. The variances and covariances of the reflectances depend on the degree of cloud variability, as well as radiative variability caused by 3‐D radiative effects (discussed in more detail in the next section). Using MODIS data, Zhang et al. (2012, hereafter Z12; see their Figure 12) showed that *r*_e_ tends to be fairly constant within the 1 km scale of a MODIS pixel but that *τ*_c_ displays considerable variation. The variance in reflectances caused by this cloud variability, combined with the nature of the MODIS forward model, means that subpixel effects actually tend to cause an overestimate of *r*_e_ (Zhang & Platnick, 2011; Zhang et al., 2016, Z12), which is in contrast to the expected result when independent retrievals are assumed. Figure 2 shows an example taken from Z12. This could explain a positive bias documented in MODIS *r*_e_ relative to in situ values by Painemal and Zuidema (2011). For *τ*_c_ an underestimate was generally found, which is consistent with the plane‐parallel albedo bias.

Z12 provide some information on the overall *r*_e_ bias from subpixel effects for a cumulus cloud case. They performed MODIS‐like retrievals upon cloud fields generated by a high‐resolution (100 m) LES after applying either 1‐D or 3‐D radiative transfer (RT). Their paper shows the differences between 2.1‐μm *r*_e_ retrievals performed on the high‐resolution reflectances and those applied to the reflectance field coarse grained to 800‐m resolution (i.e., close to MODIS resolution). When using 1‐D RT it reveals large positive subpixel biases (defined here as the coarse resolution *r*_e2.1_ minus the high‐resolution *r*_e2.1_) of up to 20 μm for the more heterogeneous pixels and smaller biases of <5 μm for the less heterogeneous pixels. Negative *τ*_c_ biases with magnitudes of up to 100% were also reported but with no delineation between low and high heterogeneities. In reality, though, 3‐D radiative transfer occurs (see the next section) and the subpixel effects are mediated by this. Z12 found lower subpixel biases for 3‐D RT than for 1‐D RT (<5 μm for less heterogeneous pixels and ≲15 μm for the more heterogeneous ones). The negative *τ*_c_ subpixel biases were mostly within 40%. The authors further find that the subpixel bias for *r*_e3.7_ is less severe and also that the difference between the two retrievals can give some quantification of the subpixel bias for *r*_e2.1_.

The results of Werner et al. (2018) also provide some information on the magnitude of the subpixel effect through the use of 30‐m resolution Advanced Spaceborne Thermal Emission and Reflection Radiometer (ASTER) data for 48 60 × 60 km stratocumulus scenes taken off the coast of California. Retrievals were performed at both 30‐m resolution and after averaging to 960 m, with the differences (high minus low resolution) indicating the subpixel bias. The first percentile, median, and 99th percentile of the biases were −0.6, −0.1, and 0.01 (−3.9%, −0.5%, and +0.4%) for *τ*_c_, and −0.02, 0.1, and 0.7 μm (−0.2%, +0.5%, and +4.7%) for *r*_e_. The results suggest that a lot of points had a relatively low bias, but the fact that the *r*_e_ bias distribution is skewed toward positive values is important given the highly nonlinear effect of *r*_e_ upon *N*_d_. The *τ*_c_ biases were skewed toward negative values in agreement with the negative bias demonstrated in Z12. Since the high‐resolution retrievals may be subject to 3‐D radiative effects (see section 2.4.2) the subpixel biases for *τ*_c_ and *r*_e_ are likely to be underestimated. Likewise, the analysis was only performed on fully overcast 960‐m pixels and biases would be likely to be higher for partially cloudy pixels, which constituted a significant fraction of the scenes that were analyzed (see also Werner et al., 2016).

**Figure 3:**
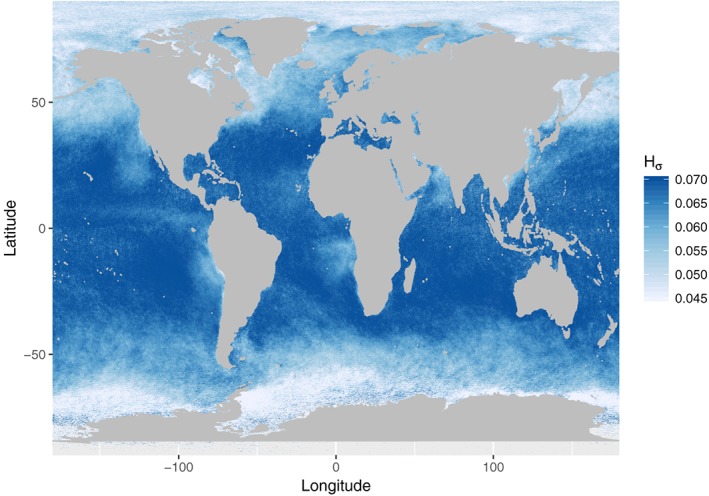
Time‐mean H_σ_ for the visible 0.86‐μm channel compiled from MODerate Imaging Spectroradiometer (MODIS) Level‐2 data for the year 2008 and for single‐layer liquid clouds with cloud top temperature >0 °C only.

One practical tool for identifying inhomogeneous pixels and estimating the quality of MODIS retrievals is the heterogeneity index *H*_*σ*_ (Liang et al., 2009), which provides a measure of the variation of the 250‐m resolution reflectance measurements (i.e., the visible 0.65‐ and 0.86‐μm channels) within a 1 × 1 km^2^ pixel. This index is operationally provided in the Collection 6 MODIS products (Platnick et al., 2017), although currently only for Level‐2 data. *H*_*σ*_, along with knowledge of the degree of nonlinearity between the reflectances and retrieved quantities within the forward model (i.e., the partial derivatives mentioned earlier in this section), may also be used for a possible correction for subpixel heterogeneity issues (Zhang et al., 2016), as explained in section 4. Figure 3 shows a map of the time‐mean *H*_*σ*_ for the year 2008 compiled from MODIS Level‐2 data. Individual pixel‐level values larger than 0.1 have been removed from the data set. High values give some indication of regions where the subpixel bias is likely to be high, although variability in the SWIR channels and covariability are not included in this metric. The figure shows that lower values are obtained in the stratocumulus‐dominated regions. The very low values in the Arctic and around Antarctica, however, are likely influenced by the presence of sea ice or high SZAs and may not therefore indicate regions where retrievals are reliable. Cho et al. (2015) find that the failure rate in MODIS retrievals becomes significant for *H*_*σ*_>0.3, although with a strong dependence on viewing geometry. Such failures are likely due to a combination of subpixel heterogeneities and subpixel cloud‐free regions (see later in this section). Cho et al. (2015) also find, in agreement with Z12, that *r*_e_ retrievals obtained using the 3.7‐μm channel are less impacted by subpixel heterogeneities than when retrieved from the 2.1‐μm channel.

A related problem to subpixel variability in cloud properties is that cloud‐free regions are likely to exist within the scale of larger satellite pixel footprints (e.g., within 1 km for MODIS), whereas retrievals generally assume a fully cloudy pixel. The presence of cloud‐free regions could be considered as being similar to subgrid *τ*_c_ variability within a pixel with the cloud‐free regions having zero *τ*_c_ and thus very low visible reflectances. However, the cloud–free regions are also likely to introduce some very high SWIR reflectances too (i.e., the value corresponding to the surface).

Leahy et al. (2012) give an idea of the likely scale of this problem by using colocated satellite cloud lidar data from the Cloud‐Aerosol Lidar and Infrared Pathfinder Satellite Observations (CALIPSO; Winker et al., 2009) to provide distributions of cloud lengths. When considering all low (<3‐km altitude) marine clouds with no contamination from high‐altitude clouds it was found that clouds with lengths of less than 1 km (i.e., smaller than a MODIS pixel) accounted for a large fraction of the total observed number of clouds. However, for stratocumulus regions (where the *N*_d_ retrieval is most likely to be applied to) it was found that almost all clouds that were smaller than 4 km in size had *τ*_c_<3. Thus, since the overall *τ*_c_ value over the 1‐km region of a MODIS pixel is likely to be less than 3 if the subpixel cloud elements have *τ*_c_<3 then restricting analysis to pixels with *τ*_c_>3 allows the issue of partly cloudy pixels for stratocumulus to be circumvented.

Coakley et al. (2005) and Hayes et al. (2010) describe a retrieval method that estimates the degree of partial cloudiness within a MODIS pixel and perform a retrieval that attempts to correct for it. On average, though, the relative variabilities in *τ*_c_ and *r*_e_ retrieved using the partially cloudy pixel retrieval were similar to those using the standard MODIS retrievals even in pixels identified as being partially cloudy by CALIPSO, suggesting that either the retrieval does not account for all of the biases caused by subpixel variability or that the partial cloudiness effect is, on average, not large for stratocumulus clouds. However, for overcast pixels within broken cloud regions, or for partially cloudy pixels, the partially cloudy pixel retrieval produced values for dln(*r*_*e*_)/dln(*τ*) that were closer to the value of 0.2 expected for adiabatic clouds than for the standard MODIS retrievals indicating some improvement. Cloud top temperatures within partly cloudy pixels from the partly cloudy‐pixel retrieval also produced a closer match to those derived from CALIPSO than the standard MOD06 retrieval.

The strong sensitivity of *N*_*d*_ to *r*_e_ through equation (11) implies that the subpixel effect tends to lead to an underestimation of *N*_d_ and that the underestimate is likely greater in highly heterogeneous cloud fields. An estimate of the likely overall subpixel error from the literature is lacking, although the results of Z12 suggest that the bulk of the pixels from their LES cumulus cases with realistic 3D radiative transfer had a subpixel bias of less than 15%. However, this is based on modeled clouds and not real clouds and only represents one case study; results from additional cases and observational estimates are needed.

#### 2.4.2 Resolved 3‐D Radiative Effects

Section 2.4.1 discussed retrieval errors due to real‐world variability of *τ*_c_ and *r*_e_, as well as apparent variability of reflectances within the scale of the satellite pixel (1 km in the case of MODIS for viewing at nadir) where the true mean *τ*_c_ and *r*_e_ values are not obtained when performing retrievals on the pixel–averaged reflectances. Here we discuss errors due to resolved (i.e., occurring at scales larger than the pixel size) deviations of the reflectances from that which would be expected from a pixel that was isolated in space (or more specifically a horizontally uniform pixel that was infinite in extent); that is, a breakdown of the plane‐parallel (PP) independent pixel approximation (Cahalan et al., 1994). This occurs when there is a net horizontal flux of photons into or out of the pixel boundaries, often called “3‐D radiative effects.” We formally define the deviations here as

(15) $$\begin{array}{ccc} \Delta R_{\text{vis}} & =R_{\text{3D vis}}-R_{\text{PP vis}} & \\ \Delta R_{\text{SWIR}} & =R_{\text{3D SWIR}}-R_{\text{PP SWIR}}, \end{array}$$

where the “3‐D” subscript refers to the actual reflectances received and the “PP” subscript refers to the PP independent pixel approximation reflectances.

There are several possible causes of these deviations, but all arise from some kind of cloud heterogeneity. Vertical variability of cloud top height can give rise to shadows (and thus negative Δ*R* values) upon regions on the side opposite to the Sun due to a reduction in illumination, with the illuminated side producing positive Δ*R* values. These effects are more prominent when the Sun is low in the sky (i.e., a high SZA). However, even without cloud top height variation Δ*R* deviations can occur when there is internal cloud variability via the so‐called “channeling” effect (Cahalan & Snider, 1989; Cannon, 1970; A. Davis, et al., 1990; Loeb et al., 1997), whereby incoming radiation gets preferentially scattered horizontally from denser portions of the cloud into the less dense regions leading to lower reflectances and hence *τ*_c_ retrievals. In contrast to shadows, such effects can occur even when the Sun is overhead. Both cloud top height variability and internal variability act to increase channeling under an overhead Sun, with the relative impact of the internal variability becoming larger at larger spatial scales (Loeb et al., 1997; Várnai & Davies, 1999; Zuidema & Evans, 1998), reflecting increased internal variability in both *τ*_c_ and *r*_e_.

When retrievals are performed on the 3‐D reflectances the overall mean *τ*_c_ and *r*_e_ values over a number of pixels are not the same as the true mean values. M06 showed that (in a similar manner as for the subpixel effects except in reverse) this arises due to the nonlinearity of the relationships between *τ*_c_ and *R*_vis_, and between *r*_e_ and *R*_SWIR_, and predicted an overestimate of both *τ*_c_ and *r*_e_. However, it should be reiterated that those theoretical arguments did not take into account the dual dependence of *τ*_c_ and *r*_e_ upon both *R*_vis_ and *R*_SWIR_, and it was also assumed that there would be equal and opposite contributions from positive and negative Δ*R*_vis_ and Δ*R*_SWIR_ values over the region being considered. Nevertheless, the theoretical predictions were corroborated by results where 3‐D radiative transfer and bispectral MODIS‐like retrievals were applied to cloud fields generated by LES for a SZA of 60° (i.e., fairly low Sun conditions). The LES results showed that when retrievals were performed at the native LES resolution there was a 6‐μm (60%) increase in the mean *r*_e_ for cumulus fields (67‐m resolution) and a 2‐μm (20%) increase for a stratocumulus cloud field (55‐m resolution). The bias in mean *τ*_c_ due to such resolved 3‐D effects was shown in both Zuidema and Evans (1998) and Varnái and Marshak (2001) to be +2 (13% using the mean *τ*_c_ of the former study) for an SZA of 60° and nadir viewing, at spatial resolutions of 200 m and 50 m, respectively. At lower SZA the *τ*_c_ bias becomes negative due to the above‐mentioned channeling effect; Zuidema and Evans (1998) shows the bias to be −0.7 (5%) for overhead Sun at 200‐m resolution and Varnái and Marshak (2001) indicate a similar bias of −0.5 for SZA = 15° at 250‐m resolution. Varnái and Marshak (2001) also suggest that the relative *τ*_c_ biases remain constant as *τ*_c_ increases for *τ*_c_< 20. Both of these studies show that higher biases from these effects are expected for higher spatial resolutions, which is discussed further in the next section.

#### 2.4.3 Discussion of Resolved Versus Subpixel Heterogeneity Issues for Retrievals

When moving to larger averaging scales (i.e., the scale over which reflectances are averaged before retrievals are performed, which may also occur unintentionally due to lower instrument resolution) there is some degree of cancelation of the positive and negative Δ*R* values, which mitigates the resolved heterogeneity effects. This was demonstrated in the above‐mentioned M06 LES study for *r*_e_ (see their Figure 4) and in Zuidema and Evans (1998) and Varnái and Marshak (2001) for *τ*_c_. However, the resolved heterogeneity then becomes increasingly subpixel and the subpixel heterogeneity artifacts discussed previously apply. A. Davis et al. (1997) demonstrated that for *τ*_c_ retrievals, there is a “sweet spot” resolution at which to average reflectances over in order to minimize the overall error. A difficultly is that this scale is likely to vary between cloud scenes and to depend upon cloud type and viewing geometry. Consistent with the idea of an ideal averaging scale Zuidema and Evans (1998) and Varnái and Marshak (2001) suggested that for a high SZA of 60°, positive *τ*_c_ biases reduce to ≲1 (6%) at the MODIS spatial resolution of (1 km) but then become negative at lower resolutions. M06 also observed very low *r*_e_ biases at an averaging scale of 500–900 m. However, for 800‐m retrievals Z12 found an overall positive bias in *r*_e_ due to large subpixel effects, which is inconsistent with the M06 result. A likely factor here is that the M06 study was performed at an SZA value of 60°, whereas the Z12 cases were for SZA = 20 and 50°, an idea which is consistent with the results of Zuidema and Evans (1998) albeit for *τ*_c_ rather than *r*_e_. There remains a need to resolve these discrepancies in future work and also to quantify the overall *r*_e_ biases for the cases presented in Z12 and to do this separately for the different viewing and solar geometries studied. At very high SZAs ≳ 65° retrieval errors can become high even at large spatial averaging scales; this is discussed in section 2.4.5.

Very high resolution instruments such as, for example, ASTER (15‐ to 30‐m resolution; e.g., see Werner et al., 2016), Landsat (30‐m resolution; e.g., see Oreopoulos et al., 2000), and European Space Agency (ESA) Sentinel 2 (10–20 m for visible and SWIR wavelengths, 60 m for atmospheric correction bands, Drusch et al., 2012) may prove useful for assessing subpixel variability and choosing the best averaging scale, although the high‐resolution retrievals are subject to resolved 3‐D radiative effects and it is difficult to determine the overall bias.

For stratocumulus clouds in the southeast Pacific (VOCALS campaign), a MODIS *r*_e_ overestimate of 15–20% was reported in Painemal and Zuidema (2011), 13% in King et al. (2013), and 17–30% in Min et al. (2012) for comparisons to aircraft observations. In addition, these studies tended to show a relatively low variability of the percentage biases; Min et al. (2012) indicated relative standard deviations of 15–20%. This suggests a reasonably constant systematic bias for these clouds. Following Z12, and since the VOCALS measurements were at low SZAs, subpixel heterogeneity biases are a likely cause of such differences, particularly when combined with the fact that the largest MODIS overestimates reported in King et al. (2013) occurred when drizzle drops were present, which implies large cloud heterogeneity. In section 2.4.1 an estimate of the subpixel *r*_e2.1_ bias from the cumulus case of Z12 of ≲15% was made, which is similar to the observed *r*_e_ overestimate found during VOCALS, although the clouds observed in VOCALS were more homogeneous than the cumulus transition clouds studied in Z12.

It should also be considered that sizing errors are possible from the aircraft instruments too; King et al. (2013) estimated an *r*_e_ uncertainty of at least 10% through the intercomparison of the two droplet sizing instruments flown during VOCALS, which is close to the observed MODIS *r*_e_ bias. Platnick and Valero (1995) found even larger differences of around 30% between two different in situ probe measurements of *r*_e_, although this was an older study and so may not reflect recent instrument improvements.

Other previous studies in other regions (Bréon & Doutriaux‐Boucher, 2005; Nakajima & Nakajma, 1995; Nakajima et al., 1991) have also indicated a high bias in MODIS *r*_e_ retrievals in marine stratocumulus regions; the latter suggested a bias of 2 μm (20%) with a standard deviation in the bias of 1.5 μm from comparison with the POLarization and Directionality of the Earth's Reflectances (POLDER) satellite instrument. However, in an aircraft remote sensing study of marine stratocumulus off the coast of California, Alexandrov et al. (2015) found a negligible mean difference between *r*_e_ retrievals from a polarimeter instrument (the Research Scanning Polarimeter [RSP], see section 4.3) and bispectral retrievals from a MODIS‐like instrument (the Autonomous Modular Sensor), with a correlation coefficient of 0.93 and a standard deviation of the differences of 0.68 μm. Some caveats here are that the clouds being observed were found to be very homogeneous, with an equivalent *k* value of 0.97, and thus less subject to heterogeneity‐induced biases. The fact that this was an aircraft study might also introduce differences relative to what would be observed in a satellite comparison (e.g., due to the lack of intervening atmosphere, or since a much higher observation resolution is achieved). This, along with the use of a different instrument to MODIS, may make the results less suitable for assessing MODIS biases.

*τ*_c_ biases from the VOCALS campaign were found to be small and variable although with a tendency for more positive biases. This is consistent with the low *τ*_c_ theoretical biases expected at low SZAs (Varnái & Marshak, 2001; Zuidema & Evans, 1998), although of opposite sign.

Overall, the above discussion suggests retrieval biases resulting from cloud heterogeneity of 17% (overestimate) for *r*_e_ and 5% for *τ*_c_ for stratocumulus clouds for low (<60°) SZAs for the resolution of the MODIS instrument. The error is likely to be higher for more heterogeneous trade cumulus or transition clouds. For *r*_e_, this uncertainty is mostly a bias rather than a statistical error. A measure of the variability of the bias for different cloud environments would therefore be desirable since if the mean offset was known and a correction was applied, then it would be the variability in the offset that would determine the uncertainty. Considerations similar to this are discussed in more detail in section 2.6.

#### 2.4.4 Errors in Retrieved r_e_ Due To Droplet Distribution Width and the Presence of Precipitation

Section 2.3.2 discussed the effect of the assumption of a constant DSD width (via the *k* parameter) upon *N*_d_ retrievals that arise solely due to the formulation of the *N*_d_ equation (equation (11)). However, uncertainties in *k* can also lead to uncertainties in the *r*_e_ value that is retrieved by satellite, and hence to further *N*_d_ errors; these errors are discussed in this section, along with potential effects due to precipitation.

While *N*_d_ represents the 0th moment of *n*(*r*) for a DSD, satellite instruments are sensitive to higher moments, namely, the cross section (second moment of *n*(*r*)), mass (third moment of *n*(*r*)) or the radar reflectivity factor (sixth moment of *n*(*r*)). Retrievals from such instruments are therefore very dependent on assumptions about the width and shape of *n*(*r*).

In order to retrieve *τ*_c_ and *r*_e_, the MODIS algorithm assumes a DSD of fixed shape, which is set to a modified gamma function (equation (12)). If *r*_e_ and *v*_e_ are both known, the assumed‐shape size distribution is determined and Lorenz‐Mie theory can be used to compute the cloud single‐scattering properties necessary to simulate satellite reflectances. *r*_e_ is retrieved while, due to a lack of information, *v*_e_ is set to a fixed value of 0.10 (Zhang, 2013). Using equation (13), this *v*_e_ value corresponds to *k* = 0.72. Section 2.3.2 suggests that such a *k* value is more likely to be an underestimate than an overestimate except perhaps for continental clouds.

Zhang (2013) showed that for dual‐mode DSDs, which can occur due to the development of a precipitation mode, the retrieved *r*_e_ is likely to be lower than the true overall *r*_e_ (i.e., when the true *r*_e_ takes into account both modes) and that this underestimate is worse for the 3.7‐μm *r*_e_ retrieval than for the one using the 2.1‐μm channel. However, the number of droplets in the rain mode is likely to be negligibly small compared to that in the cloud mode (e.g., see Figure 7 in Nakajima et al., 2010a) and given the large size of the rain mode it will contribute little to the overall *τ*_c_. Thus, when considering *N*_d_ retrievals a better estimate of *N*_d_ would be obtained if the *r*_e_ of only the cloud mode was retrieved. Zhang (2013) and Nakajima et al. (2010a) suggest that *r*_e_ retrievals are likely to be biased high relative to the *r*_e_ of the cloud mode in situations with dual‐mode DSDs. This would lead to an underestimate of *N*_d_. If the *k* value also gets smaller in such situations then this too would lead to an underestimate of *N*_d_ (see section 2.3.2) via equation (11) and thus these errors would reinforce each other. Also, compared to the cloud mode *r*_e_, the *r*_e2.1_ overestimate is likely to be worse than that for *r*_e3.7_. Conversely, in the situation with only a single cloud mode DSD the results of Platnick and Valero (1995), Zhang (2013), and Chang and Li (2001) indicate that an *r*_e_ underestimate will occur if that cloud mode has a wider distribution than assumed by the MODIS retrieval (i.e., *v*_e_> 0.1, or *k*< 0.72). This causes an *N*_d_ overestimate, which counteracts the underestimate from the assumption of a k value that is too large.

It should also be considered whether the presence of a rain mode may lead to the violation of some of the other assumptions for the *N*_d_ retrieval and hence *N*_d_ errors. For example, section 2.3.3 discussed the possibility that rain might lead to subadiabatic *L* profiles or a departure from the assumed constant vertical gradient of *L*. It is also possible that precipitation may also invalidate the vertically constant *N*_d_ assumption (section 2.3.5). However, Zhang et al. (2012) found that the presence of precipitation had little effect on *r*_e_, which is the quantity that is likely to have the biggest impact upon *N*_d_ uncertainty (see section 2.6), for MODIS retrievals performed on model‐generated clouds.

As a precautionary measure, it may be prudent to attempt to filter out situations with precipitation before performing *N*_d_ retrievals. This is difficult to do definitively using passive retrievals, although Rosenfeld, Fischman, et al. (2014) suggest that insignificant collision coalescence occurs when $r_{e}\;≲$ 14 μm. The CloudSat satellite cloud radar instrument (Stephens et al., 2002) can detect low drizzle rates and could also be used to filter out precipitating clouds for *N*_d_ retrievals based on the MODIS instrument on board Aqua, although its across‐track sampling width (1.4 km) is very small compared to the MODIS swath width (2,300 km).

Since the effect of the DSD width on *N*_d_ is variable depending on the presence or not of a rain mode and since there have been only a few studies looking at the effect of DSD width and precipitation in detail (and without any consideration of the impact upon *N*_d_ retrievals), we neglect these biases for the *N*_d_ retrieval with the assumption that *r*_e_ errors for the subpixel variability effect (section 2.4.1) are likely to be larger. Furthermore, the homogeneous stratocumulus clouds that are the focus of this review are likely to produce low precipitation rates or even to be nonprecipitating.

Finally, we note that information on *v*_e_ from remote sensing using polarimetric measurements may help to further characterize and constrain DSD‐related errors in the future (see section 4.3).

#### 2.4.5 Viewing Geometry

The relative positions of the Sun, the cloud being observed and the satellite, that is, the viewing geometry, can vary greatly and yet can have a large impact upon the retrieved *τ*_c_ and *r*_e_ values and therefore upon *N*_d_ estimates. Here we discuss biases as functions of the overall scattering angle (SA), which is the angle subtended by the Sun, the scattering point (e.g., the cloud or ground), and the satellite instrument as measured in the same plane; the SZA, which is the angle subtended by the Sun, the scattering point and the zenith, so that an overhead Sun has SZA = 0° ; the viewing zenith angle (VZA), which is the angle subtended by the scattering point, the satellite and the nadir; and the relative azimuth angle (RAZ), which is the angle subtended by the Sun, the scattering point and the scattered light after being projected onto a horizontal plane. The definition of RAZ is such that forward scattering corresponds to RAZ = 0° and backscattering to RAZ = 180°.

Liang et al. (2015) used the multiple view capability of the MISR (Multiangle Imaging Spectroradiometer) instrument to examine global data for a range of SAs. Collocated MODIS observations provided *r*_e_ values to use in the retrieval of *τ*_c_ by MISR (MISR cannot retrieve *r*_e_ itself). By examining the magnitude of the change in the MISR retrieved *τ*_c_ around scattering angles of 140° (so‐called rainbow scattering), they inferred and quantified positive biases in the MODIS *r*_e_ for low‐level water clouds over oceans. The midpoints of the upper and lower bounds in the zonal mean biases were found to be 3–11 μm for *r*_e1.6_ and *r*_e2.1_, and 2–7 μm for *r*_e3.7_. These estimates are for all low‐altitude liquid cloud types and so include trade cumulus and other heterogenous cloud types. Any biases would be lower for stratocumulus, which is suggested by the lower bias estimates for latitudes that are dominated by this cloud type. Also, since the bias estimate method presented in Liang et al. (2015) is novel and requires a number of assumptions (e.g., the use of 1‐D radiative transfer models), they should be treated with some caution until they are further corroborated.

Bennartz and Rausch (2017) examined issues related to the scattering angle for MODIS Collection 6 retrievals of *r*_e3.7_, *τ*_c_ and *N*_d_ (calculated with methods similar to those described in section 2.1). Global annual averages of *r*_e3.7_ and *τ*_c_ showed substantial increases for SA < 90° with differences of up to Δ*r*_e3.7_=3 μm and Δ*τ*_c_=30 compared to at SAs between 100 and 170°. This led to relatively small changes in *N*_d_, though, due to cancelation effects. There was also a pronounced spike for *r*_e3.7_ centered at SAs of around 175°, with a positive difference of between 3 and 6 μm depending on the particular data set examined. This had a larger effect on *N*_d_ (with a reduction of up to around 40 cm^−3^, 50%) than the low SA differences since only *r*_e_ was affected (and not *τ*_c_). The suggestion was made that these errors are caused by assumptions in the retrieval process. Data density at these particular scattering angles was, however, reasonably low and also potential preferential geographical variation as a function of scattering angle was not examined. There are also correlations between the scattering angle and the other viewing geometry angles (VZA, RAZ, and SZA) and a given SA can come about through various combinations of these other angles, so it is also useful to examine uncertainties as separate functions of these. For example, the same SA could occur at two different SZA values, which would produce different degrees of cloud radiative heterogeneity.

There have been several studies that have examined the effect of SZA upon MODIS‐like *τ*_c_ retrievals (e.g., Kato & Marshak, 2009), with the conclusion that *τ*_c_ is likely to be overestimated at high SZA due to enhanced upward scattering of light by realistic heterogeneous clouds relative to the plane‐parallel clouds used for most forward models. Várnai and Davies (1999) showed that cloud top heterogeneity is likely to contribute more to this effect than internal cloud variability. This was examined in detail by Zuidema and Evans (1998) using cloud fields reconstructed from vertically resolved cloud radar fields. They found that cloud top heterogeneities could lead to an overestimate in *τ*_c_ retrievals even at the 1 km scale, exceeding the underestimate generated by internal photon transport, at SZA = 60° . Grosvenor and Wood (2014) estimated biases in *r*_e_, *τ*_c_, and *N*_d_ for MODIS data at high SZA by using the variation of SZA throughout the diurnal cycle to overcome the problem of covariance of SZA (and potentially *r*_e_, *τ*_c_, and *N*_d_) with latitude. They found positive biases in *τ*_c_ of around 70% at SZA values close to 80° and smaller negative biases in *r*_e_ of 5% for the 2.1‐μm retrieval and 7% for 3.7 μm. Thus, both the *τ*_c_ and *r*_e_ biases acted to increase *N*_d_, which was positively biased by around 50%. Generally, the *τ*_c_ biases contributed more to the *N*_d_ error than *r*_e_ biases, except for clouds with very heterogeneous cloud tops when the contributions were roughly equal. The *τ*_c_, *r*_e_, and *N*_d_ biases were observed to occur at SZA > 65° (see also Várnai & Davies, 1999). SZA values such as these will be prevalent in the winter season for middle to high latitudes, and also for retrievals obtained near to dawn and dusk. The latter can occur for geostationary satellites and also for polar orbiting satellites at high latitudes where several overpasses per day occur for a given location. In summer, the biases due to diurnal sampling are diluted when averaging over the whole day, but care should be taken when examining individual retrievals. Following Grosvenor and Wood (2014), the uncertainty assessment is that *N*_d_ error is negligible for SZA < 65° , about 40% for SZA ≈ 70° , and 60% for SZA ≈ 80°.

A number of studies have examined the consistency between satellite observations from the different view angles of the same cloud pixels afforded by the MISR satellite in order to assess the validity of the PP retrieval assumptions (Di Girolamo et al., 2010; Horváth, 2004; Liang et al., 2009). The approach was to compute (assuming PP clouds) 0.86‐μm reflectances for the different MISR view angles based upon the nadir view *τ*_c_ from MISR and *r*_e_ from collocated MODIS retrievals. These were then compared to the actual reflectances at the different view angles as measured by MISR to calculate the root mean square (RMS) of the relative differences (denoted as *m*_BRF_). *m*_BRF_ should theoretically be zero since the same cloud is being viewed but at multiple angles. Di Girolamo et al. (2010) showed that for stratocumulus for most examined situations, ≳80% of the data points have *m*_BRF_ values of <5%. Liang et al. (2009) also looked at the standard deviation of the retrieved *τ*_c_ across the different MISR view angles (*m*_*τ*_) for only one swath. They found that data points with *m*_*τ*_ values of <10% occurred 85% of the time. It was also shown in Liang et al. (2009) and Di Girolamo et al. (2010) that *m*_BRF_ and *m*_*τ*_ increased as a function of the 0.86‐μm reflectance heterogeneity parameter (*H*_*σ*_; calculated using MISR 275‐m resolution reflectances) suggesting that the latter is a useful measure of the degree to which PP assumptions are invalid.

Kato and Marshak (2009), Liang and Girolamo (2013), and Varnai and Marshak (2007) suggest that larger *τ*_c_ values are expected with increasing VZA for low SZAs (below 40°), although it seems that the difference between nadir *τ*_c_ and that at VZA < 60° is <10%. Thus, most of the MODIS data that is sampled has these relatively low VZA biases since the maximum VZA for MODIS is 66°. Additionally, the bias was found to be worse for heterogeneous clouds than for homogenous ones in Liang and Girolamo (2013) and Varnai and Marshak (2007). However, the study of Maddux et al. (2010) suggested underestimates (relative to nadir VZA) of *τ*_c_ of up to 25%, albeit at very high VZAs (>60°); the results of Liang and Girolamo (2013) identify some mechanisms by which a negative *τ*_c_ bias is possible. Liang et al. (2015) also reported nonmonotonic variations of *τ*_c_ with VZA, which is consistent with the lack of agreement between Maddux et al. (2010) and the other studies mentioned above. Liang and Girolamo (2013) used combined MISR and MODIS observations to study how *τ*_c_ varies with VZA, while also taking into account the effect of RAZ and SZA. They found that RAZ appears to only be relevant at higher SZAs (>40°), although this may also reflect a lack of sampling of forward and backward scattering RAZ angles at lower latitudes for polar orbiting instruments and thus may not be the case for all instruments. At high SZA the dependence of *τ*_c_ upon VZA becomes more complicated with both negative and positive biases seemingly possible, depending on RAZ.

For *r*_e_ a positive increase of around 15% for stratocumulus was reported for high VZAs by Maddux et al. (2010); again, though the results apply only for VZAs > 60°. The results of Liang et al. (2015) also show an *r*_e_ increase but suggest that it only occurs for VZAs >  55°. Taken alone, an *r*_e_ overestimate of 15% causes an *N*_d_ underestimate of around 40% based upon equation (11) for such high VZAs (and at low SZA). Since there are conflicting conclusions regarding *τ*_c_ biases at high VZAs we do not include them in our calculation. Based on the above, *N*_d_ biases at lower VZAs are likely to be negligible and so it is prudent to restrict the use of *N*_d_ retrievals to VZAs of less than 55°, which does not remove a lot of data for instruments such as MODIS.

The above studies (with the exception of Kato & Marshak, 2009) examine *τ*_c_ and *r*_e_ variations relative to nadir but do not quantify any potential nadir biases. Such biases are discussed in sections 2.4, 2.4.2, and 2.4.3.

#### 2.4.6 Upper Level Layers of Thin Cloud and Aerosol

Layers of thin cloud and aerosol overlying low clouds extinguish radiation that can be erroneously attributed to the low clouds. One such example occurs in the southeast Atlantic, where low cloud decks have been observed to reach their maximum extent at the same time (September) as equally extensive smoke layers above the clouds (Adebiyi et al., 2015). The spectrally dependent aerosol extinction is weighted more strongly to the shorter visible (and ultraviolet) wavelengths. As such, the extinction influences optical depth retrievals, (conventionally done at 0.86 μm over the ocean), more than *r*_e_ retrievals. An evaluation of MODIS clouds products in the Southeast Atlantic stratoculumus regions has shown that the presence of absorbing aerosol can reduce the retrieved *τ*_c_ by approximately 20%, but only affects the *r*_e_ retrieval to the extent that the *τ*_c_ retrieval is impaired (Haywood et al., 2004; Meyer et al., 2013) due to the bispectral dependence of the retrieved *τ*_c_ and *r*_e_, as discussed in section 2.4.1. Thus, although *N*_d_ is more sensitive to errors in *r*_e_ than *τ*_c_, the effect of the aerosol above the cloud is to decrease the retrieved *N*_d_ (Bennartz & Harshvardhan, 2007). Flags exist in the MODIS standard products for identifying such cases.

Overlap of liquid clouds by ice clouds has been shown from active instrumentation to occur in about 25% of sampled cases (Sourdeval et al., 2016, see also Heidinger and Pavolonis, 2005, and Joiner et al., 2010). This study and also Christensen et al. (2013) showed a strong geographical dependence for overlap with the highest rates in the midlatitude storm track regions, as well as the stratocumulus regions off the coast of California and off the west coast of southern Africa. Despite significant efforts made to detect multilayer situations (Wind et al., 2010), their proper treatment remains an important challenge for retrieval methods based on passive measurements, which for practical reasons often consider the atmospheric column to be composed of a single cloud layer of liquid or ice phase. This single‐layer approximation (SLA) can have strong consequences for *τ*_c_ and *r*_e_ retrievals (S. Davis et al., 2009; Sourdeval et al., 2013). For multilayer situations, this implies that the observed contribution of the ice cloud to the upwelling reflectance is mistakenly attributed to the liquid layer. This leads to an increase in *R*_vis_ due to scattering by the ice layer and a decrease in *R*_SWIR_ due to additional absorption. The consequence for liquid cloud retrievals is an overestimation of *τ*_c_ and/or *r*_e_ (Sourdeval et al., 2016). The cloud optical depth, however, is less impacted than *r*_e_ because of the lesser sensitivity of the visible wavelengths to ice clouds in comparison to the shortwave infrared (Yang et al., 2001).

**Figure 4:**
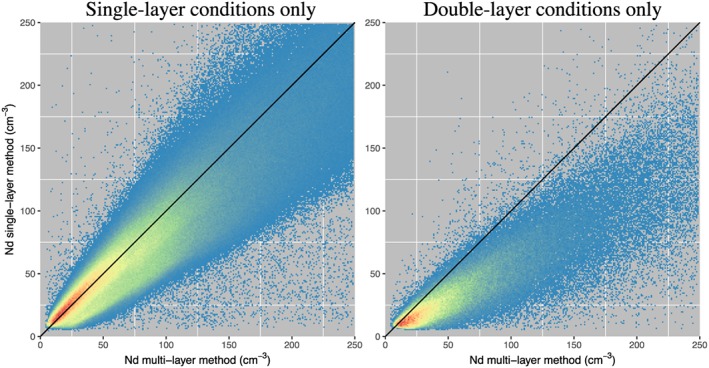
Density scatterplots comparing 1 year of global oceanic estimates of N_d_ obtained from the multilayer method (x‐axis) by Sourdeval et al. (2016) to those of MODerate Imaging Spectroradiometer Collection 5 (y‐axis) in (a) single‐layer and (b) double‐layer conditions.

As a consequence, an underestimation of *N*_d_ is expected in multilayer conditions. Figure 4 demonstrates this for *N*_d_ estimates from MODIS in comparison to those from a method that simultaneously retrieves ice cloud properties (Sourdeval et al., 2014). It is worth noting that due to an a priori choice of the cloud phase based on auxiliary information (e.g., Marchant et al., 2016), it remains unlikely that liquid cloud retrievals are provided in the case of an ice cloud that is very thick. The bias in *N*_d_ therefore is reduced to a factor of about 2 (Figure 4). However, the SLA also implies that few *N*_d_ retrievals are provided in regions where ice clouds are optically thick, which implies a bias in global climatologies.

As an uncertainty estimate for the current *N*_d_ product, Figure 4 is evaluated to find a relative error in *N*_d_ of 40% in cases of overlying layers of clouds/aerosols.

#### 2.4.7 Instrument, Surface Albedo, and Atmospheric Correction Uncertainties

Uncertainties related to instrumental errors or the accuracy of nonretrieved parameters of the forward model used to simulate reflectances can be large, depending on the sensitivity of the measurements to *τ*_c_ and *r*_e_, and thus vary with cloud *τ*_c_ and *r*_e_. Such errors include those due to instrument calibration/modeling errors, surface albedo, and atmospheric corrections, which propagate through to *τ*_c_ and *r*_e_ errors and are accounted for in the pixel‐level uncertainty estimates of MODIS for example (Hubanks et al., 2016; Platnick et al., 2017; Xiong & Barnes, 2006). Note, however, that the (often likely larger) errors due to subpixel and 3‐D radiative effects (see sections 2.4.1 and 2.4.2) are not included in these estimates.

Platnick et al. (2017, their Figure 14) show the MODIS Collection 6 pixel‐level uncertainty contributions from the various terms for a single land scene (granule) over the central United States. For *τ*_c_ the total uncertainty is ≲ 8% and the instrument measurement error dominates, except for $\tau_{c}≲5$ when the surface albedo uncertainty dominates; the *τ*_c_ error approaches 20% for $\tau_{c}\;≲1$. Surface albedo is likely to make a larger contribution at lower *τ*_c_ values because for thin clouds more light that has reflected from the surface will be received (Rosenfeld et al., 2004). For *r*_e3.7_ the total uncertainties were slightly less than for *r*_e2.1_ but in both cases were ≲10%, except for when $r_{e}\;≲\;6$ μm. For *r*_e2.1_ the instrument uncertainty dominates for $r_{e}\;≲\;21$ μm above which the surface albedo uncertainty dominates. For *r*_e3.7_ the surface albedo uncertainty contribution is much smaller and errors are dominated by the instrument uncertainties (including that due to the thermal emission correction necessary for *r*_e3.7_ retrievals), effective variance errors and atmospheric correction errors.

Since surface albedo errors can be large, it is worth discussing them further, although we note that the uncertainties examined above in Platnick et al. (2017) were over the land, where MODIS surface albedo uncertainties are likely to be much higher than over the oceans (Bréon & Doutriaux‐Boucher, 2005; King et al., 2004; Rosenfeld et al., 2004) since the surface albedo over land is much more variable than over the ocean. In addition, Platnick et al. (2003) point out that cloud masking is more difficult over nonvegetated surfaces, transitional areas between desert and vegetated surfaces, and above high‐altitude regions. MODIS retrievals use a different visible channel for different surface types with the aim of minimizing the surface reflectance; the 0.65‐μm channel is used over land, 0.86 μm over the ocean and 1.2 μm over bright snow/sea ice surfaces (Platnick et al., 2003). For Collection 6 of MODIS an ocean surface albedo parameterization based on Cox and Munk (1954) has been implemented (Platnick et al., 2017), which takes into account the effect of wind speed on the ocean albedo. Collection 5 retrievals assumed a spectrally flat Lambertian surface for the ocean with an albedo of 0.05. Other improvements to the handling of the surface reflectance in Collection 6 (see Platnick et al., 2017) include a new surface spectral albedo data set derived from dynamic 8‐day sampling of MODIS data (previously Collection 5 used a 5‐year surface albedo climatology, see Platnick et al., 2015) and the inclusion of land spectral emissivities that are consistent with the cloud top property algorithm. Rausch et al. (2017) showed that for one MODIS ocean scene Collection 6 *r*_e_ was up to 1 μm lower than that from Collection 5 for optically thin ( $\tau_{c}≲$ 2–3) clouds, which may be due to the surface albedo, although other changes between Collections 5 and 6 have also been made. However, Zhang and Platnick (2011) found little effect upon *r*_e3.7_ versus *r*_e2.1_ differences from the implementation of the Cox and Munk (1954) scheme for global marine liquid clouds. Since larger effects from the surface treatment for *r*_e2.1_ are expected compared to *r*_e3.7_ (Platnick et al., 2017; Rosenfeld et al., 2004), this indicates that for marine stratocumulus the impact of surface uncertainties is likely to be small, except perhaps for optically thin clouds.

Surface albedo uncertainties are also likely to be particularly problematic over ice and sea ice surfaces (King et al., 2004; Platnick et al., 2001), so that *N*_d_ estimates based upon the standard 2.1 or 3.7 μm plus 0.86 μm or 1.2‐μm channels should probably be avoided for such surface types. However, the latter papers suggest a dual‐channel retrieval method based upon the combination of the 1.6‐ and 2.1‐μm channels that may improve this situation; this retrieval for *τ*_c_ and *r*_e_ is provided separately in the MODIS products.

Using an optimal estimation approach, Sourdeval et al. (2014, see their Figure 5) showed that under optimal conditions (single layer with *τ*_c_>5, low SZA and ignoring 3D and heterogeneity effects), the accuracy of *τ*_c_ and *r*_e_ retrievals is expected to be better than 10% and 25%, respectively. Taken alone these uncertainties would lead to an error in *N*_d_ of 63% (see equation (16) in section 2.6). The *r*_e_ uncertainty found in Sourdeval et al. (2014) decreased to better than 10% when *τ*_*c*_>10 this implies an *N*_d_ uncertainty of <25% (when combined with a 10% *τ*_c_ error), assuming no other errors.

In Bennartz and Rausch (2017, see their Figure 8a) the daily Collection 6 pixel‐level uncertainties in *τ*_c_ and *r*_e_ were used to calculate the monthly mean pixel‐level *N*_d_ uncertainty, which was found to be around 30% for the stratocumulus regions. Since the mean optical depth of the main stratocumulus regions vary between 9 and 19 (Grosvenor et al., 2018), this is in approximate agreement with the results of Sourdeval et al. (2014). Thus, for the instrumental uncertainty estimates in this paper we use an uncertainty of 10% for both, *r*_e_ and *τ*_c_.

### 2.5 Validation of N_d_ Retrievals Using Aircraft Observations

Current *N*_d_ products from satellite have seen only sparse evaluation using reference measurements or assessments despite such evaluations being immensely useful for diagnosing and potentially correcting for the uncertainties described in the previous sections.

In situ measurements have demonstrated some skill of the satellite *N*_d_ retrievals over the southeast Pacific; Painemal and Zuidema (2011) showed a negligible overall *N*_d_ bias and that the bias had a low variability. It should be cautioned, however, that the good agreement between the MODIS and the in situ *N*_d_ in Painemal and Zuidema (2011) also reflects fortuitious error cancelation. A systematic overestimate in the MODIS *r*_e_ was compensated by neglecting subadiabaticity, which was shown from aircraft observations to be low for this region, and by a consistent narrowing with height of the DSDs that was not accounted for a priori. Different, equally plausible choices for such parameters within the *N*_d_ equation (equation (11)) can easily differ by 20% (e.g., George & Wood, 2010), highlighting the dependency on cloud characteristics that vary regionally (e.g., in contrast to the southeast Pacific, the stratocumulus in the north Atlantic is just as likely to exhibit droplet spectra broadening with height, as it is a narrowing Brenguier et al., 2011). However, the fact that the *N*_d_ bias was consistently small for several cloud profiles suggests that the uncertainties in question were systematically rather than randomly biased, at least for the (relatively few) clouds that were sampled. This suggests that a better characterization of these systematic biases could greatly reduce *N*_d_ uncertainties. Thus, in situ assessments should ideally be performed within a wide range of cloud regimes, of which the southeast Pacific represents but one sample in the phase space of *r*_e_ biases, adiabaticity, and droplet distribution width.

Section 3 describes and compares two long‐term (2003–2015) *N*_d_ data sets that have been presented in the literature; one data set is described in Bennartz and Rausch (2017, hereafter denoted BR17) and the other is (Grosvenor & Wood, 2018) based on the methods described in Grosvenor and Wood (2014, denoted GW14) and Grosvenor et al. (2018). There has also been some validation of these data sets against in situ observations. BR17 shows comparisons to the *N*_d_ from the aircraft profiles presented in Painemal and Zuidema (2011). As in Painemal and Zuidema (2011) high correlations (≥ 0.94) and low biases (<10%) between the satellite and aircraft *N*_d_ were found, regardless of whether the nearest 1‐km pixel, or the averages over 21 × 21, or 51 × 51 pixels were used. The root mean square error, biases, and correlations were slightly worse when data were filtered to exclude pixels which did not satisfy *r*_e1.6_<*r*_e2.1_<*r*_e3.7_ indicating that this screening may actually introduce a low *N*_d_ bias (see section 3 for more details). The GW14 *N*_d_ data set is compared against observations in McCoy et al. (2018). Flight leg average aircraft *N*_d_ values were compared with those from satellite averages over the nearest 3 × 3° regions and over the nearest 3‐day time period. Thus, the comparison is over larger scales than the pixel‐level comparisons of Painemal and Zuidema (2011) and BR17. However, data from many flight campaigns in several locations are used: the southeast Pacific (VOCALS campaign as in Painemal and Zuidema, 2011), the Antarctic Peninsula (Orographic Flows and the Climate of the Antarctic Peninsula campaign), off the California coast (Marine Stratus/Stratocumulus Experiment, Cloud Systems Evolution in the Trades and Cloud‐Aerosol Research in the Marine Atmosphere campaigns), and Northern China near Beijing and Tianjin. This represents a fairly large range of conditions, including some more cumuliform clouds, although the data are dominated by VOCALS data. The results showed an overall correlation coefficient of 0.68 and the binned mean values over all campaigns showed agreement with MODIS within the standard error. This represents poorer correlation than seen in Painemal and Zuidema (2011) and BR17, and some large errors were observed for individual flight leg comparisons (up to ∼200 cm^−3^). This may indicate the difficulties faced for satellite *N*_d_ retrievals in nonstratocumulus clouds. However, part of the lack of agreement is likely due to the less strict spatial and temporal colocation of the MODIS and aircraft data.

**Figure 5:**
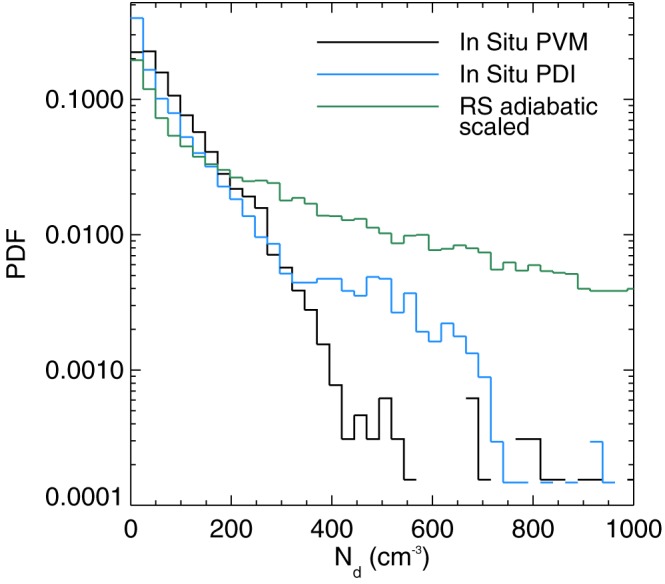
Comparison between in situ (helicopterborne Airborne Cloud Turbulence Observation System, ACTOS) and remote sensing (helicopterborne Spectral Modular Airborne Radiation measurements sysTem above cloud, collocated with ACTOS platform) N_d_ for flights trade wind cumulus clouds near in Barbados. In situ measurements are shown for both the Phase‐Doppler Interferometer (PDI) and the Particle Volume Monitor (PVM) instruments.

In situ measurements of *N*_d_ within trade wind cumuli were performed during the Clouds, Aerosol, Radiation, and tuRbulence in the trade wind regime over BArbados campaign (Siebert et al., 2013) near Barbados and compared to *N*_d_ estimated using equation (11) with *τ*_c_ and *r*_e_ retrieved at ∼5 m horizontal resolution by the helicopterborne Spectral Modular Airborne Radiation measurements sysTem (SMART‐HELIOS) remote sensing system (Werner et al., 2013). SMART‐HELIOS measures spectral reflected radiances at wavelengths between 350 and 2,100 nm and retrieves *τ*_c_ and *r*_e_ using similar principles to that employed by MODIS, although with a more sophisticated choice of channel combination and at much higher spatial resolution. The concurrent in situ measurements were performed by the Phase‐Doppler Interferometer, which measures individual droplet sizes and velocities. It was installed on the Airborne Cloud Turbulence Observation System (ACTOS, Siebert et al., 2006), which was lowered into the cloud from the same helicopter from which SMART‐HELIOS was operating, thus providing closely collocated observations from 140 m below the remote sensing instrument. Additionally, the Particle Volume Monitor in situ instrument was concurrently used, which measures Liquid Water Content (*L*) and bulk particle surface area. Figure 5 shows Probability Density Functions (PDFs) of the *N*_d_ showing good agreement between all of the instruments for *N*_d_ values between 50 and 250 cm^−3^. However, the remote sensing observations underestimated the frequencies of *N*_d_ values below this range and overestimated the frequencies at higher *N*_d_ values. This suggests either a tendency to underestimate *r*_e_ or overestimate *τ*_c_ at the tails of the distributions or that the values chosen for *k* and *f*_ad_ and/or the other assumptions inherent in equation (11) may be erroneous in some circumstances. It should also be considered that the ACTOS probe observed *N*_d_ at the very top of the cloud layer, whereas the SMART‐HELIOS instrument would observe values that are representative of deeper into the cloud. This may lead to some overestimate of the latter relative to ACTOS due to cloud evaporation at cloud top. There is also instrumental uncertainty for the in situ instruments, as exemplified by the disagreement at *N*_d_ higher than ≳350 cm^−3^.

Given the sparseness of these validation studies, in the following, we opt for a bottom‐up quantification of the overall uncertainty, rather than extrapolating the aircraft results to the global scale.

### 2.6 Overall Error Estimation

The contributions of input parameters to the relative error budget of *N*_d_ retrievals have been discussed in several stratocumulus studies (e.g., Bennartz, 2007; Janssen et al., 2011; Merk et al., 2016). Assuming the errors are normally distributed and random, the relative contributions to the uncertainty of *N*_d_ can be determined through Gaussian error propagation. For each of the input parameters in equation (11), their contribution to the overall error budget is expressed as

(16) $$\begin{array}{ccc} {\left|\frac{\partial N_{d}}{N_{d}}\right|}^{2} & ={\left|\frac{1}{2}\frac{\partial c_{w}}{c_{w}}\right|}^{2}+{\left|\frac{1}{2}\frac{\partial f_{\text{ad}}}{f_{\text{ad}}}\right|}^{2}+{\left|\frac{1}{2}\frac{\partial \tau_{c}}{\tau_{c}}\right|}^{2}+{\left|\frac{\mathrm{\partial k}}{k}\right|}^{2} & \\ & \;+{\left|\frac{5}{2}\frac{\partial r_{e}}{r_{e}}\right|}^{2}+{\left|\frac{\partial N_{d}}{N_{d}}\right|}_{\text{other}}^{2} \end{array}$$

Here the ${\frac{\partial N_{d}}{N_{d}}}_{\text{other}}$ term represents other error sources of *N*_d_ that are not related to the listed parameters. In contrast to the error budget of Bennartz (2007), *c*_w_ and *f*_ad_ are expressed as separate parameters. From the above equation, it is apparent that the *N*_d_ uncertainty, *∂* *N*_d_, is more sensitive to relative changes in *r*_e_ compared to the other parameters. For each term, the potential systematic and random contributions to *N*_d_, as discussed in the previous sections, are summarized below. The relative contributions between studies can differ significantly depending upon the underlying assumptions for parameters such as *k* and *f*_ad_.

**Figure 6:**
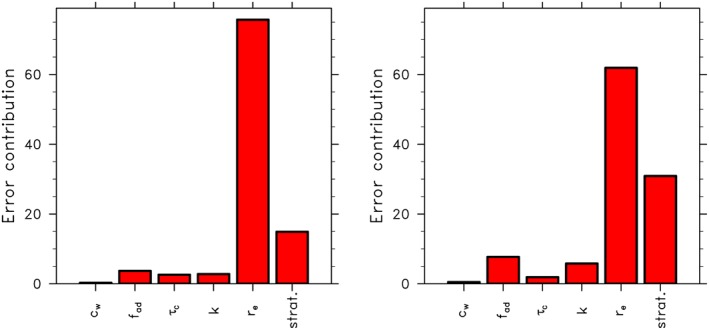
Relative contributions (%) to the percentage error squared from each of the terms on the right‐hand side of equation (16), which are associated with the various parameters that affect N_d_. Contributions are expressed as a percentage of the overall percentage squared value. Estimates are given for the pixel level where instrument uncertainties are included for τ_c_ and r_e_ (left; total value = 6,022.5), and for averages over 1° ×1° regions where they are assumed to become negligible due to being random errors (right; total value = 3,172.5). “strat.” refers to the vertical stratification uncertainty.

The uncertainty estimates are 8% for *c*_w_ (section 2.3.4), 13% for *k* (section 2.3.2), and 30% for *f*_ad_ (section 2.3.3). Due to resolved and unresolved heterogeneity, an uncertainty in *r*_e_ of 17% was assessed in section 2.4.3 and that due to instrument uncertainty was estimated as 10% (section 2.4.7) giving an overall error of 27%. Uncertainties in *τ*_c_ due to heterogeneity (section 2.4.5), viewing geometry (section 2.4.3), and instrument uncertainty (2.4.7) were assessed at 5%, 10%, and 10%, respectively, giving an overall error of 25%. A further 30% uncertainty in *N*_d_ arises from inconsistencies in the model for vertical stratification (section 2.3.1). The total uncertainty is thus ${\left[\frac{1}{4}(8{\text{\%}}^{2}+30{\text{\%}}^{2}+25{\text{\%}}^{2})+13{\text{\%}}^{2}+\frac{25}{4}\;27{\text{\%}}^{2}+30{\text{\%}}^{2}\right]}^{\frac{1}{2}}=78\text{\%}$. It is worse still in cases of large SZA (additional 40% to 60% uncertainty for SZA > 65° ) and in case of overlying cirrus or aerosol (additional 40%). Figure 6 shows the relative contributions to the percentage error squared from each term on the right‐hand side of equation (16). It is clear that *r*_e_ errors are by far the dominant source of uncertainty from our estimates with errors due to the vertical inconsistencies, *f*_ad_ and *k* being the second most important group. Errors in the other terms are unlikely to have much relative influence. Therefore, improvements in *r*_e_ uncertainty characterization are the most beneficial in terms of improving *N*_d_ accuracy.

The very high pixel‐level error estimated above, while likely an upper limit, suggests that small‐scale *N*_d_ retrievals are of limited utility. However, if any of the uncertainties mentioned above are random then the error in their mean values will be reduced by a factor of $\sqrt{N}$ for *N* values. Commonly, satellite quantities are obtained for 1 × 1° regions, which for MODIS 1‐km pixels equates to 111 × 111 pixels (at nadir). Thus, aggregated uncertainties for random errors would be reduced by more than a factor of 100 making them negligible. However, many of the uncertainties for pixels within such 1 × 1° regions may be correlated due to the cloud conditions being similar. It seems likely, though, that the instrument uncertainties (section 2.4.7) are uncorrelated since a major uncertainty contribution comes from instrumental noise. As such, we also calculate an uncertainty estimate for 1 × 1° regions that ignores the instrumental error and so reduces the overall *τ*_c_ and *r*_e_ errors to 15 and 17%, respectively. The propagated *N*_d_ error is then 56%. Figure 6 shows that *r*_e_ errors still dominate in this case. The large difference between the uncertainty estimates suggests the need for more studies into whether the errors listed above are correlated or not for larger regions (such as 1 × 1°) of a cloud field.

An alternative method of estimating *N*_d_ errors was presented in Bennartz and Rausch (2017; their Figure 8) who showed monthly standard deviations in *N*_d_ of around 20–40% for the year 2008 for subtropical stratocumulus regions. These estimates were based upon pixel‐level *N*_d_ values that were used to calculate the daily variance over 1×1° regions. Monthly values for the standard deviation of *N*_d_ were then calculated from the square root of the time mean of the daily variances. They make the argument that this provides an upper limit for random errors since the variability will include both real *N*_d_ variations and those due to uncertainties. However, since systematic errors and those from parameters for which a constant value is chosen (i.e., *f*_ad_ and *k*) will not affect *N*_d_ variability, the true uncertainty may be much higher, as suggested by the uncertainty assessment provided above.

## 3 Current N_d_ Data Sets and Intercomparisons

In this section we examine in detail two of the MODIS *N*_d_ data sets that have been presented in the literature, as well as one that is based upon a new cloud retrieval for the Advanced Along‐Track Scanning Radiometer (AATSR) satellite instrument, in order to show the main features of satellite‐derived *N*_d_ and to discuss some of the issues and choices that arise when compiling such a data set. The differences between data sets also give some idea of the uncertainties in them.

### 3.1 N_d_ Satellite Intercomparison: Impact of Filtering Assumptions

Satellite‐derived monthly average *N*_d_ from 2003 to 2015 from the data set from Bennartz and Rausch (2017, hereafter denoted BR17) is compared to a data set (Grosvenor & Wood, 2018) for the same time period based on the methods from Grosvenor et al. (2018), which represented some modifications to the methods described in Grosvenor and Wood (2014). However, the correction for the vertical penetration depth bias (see section 2.3.1) proposed in Grosvenor et al. (2018) is not applied to the latter data set here. This data set is denoted as GW14 and excludes 1×1° data points with mean SZA > 65°, mean cloud top heights greater than 3.2 km, and liquid cloud fractions less than 80%. The methodology used here differs slightly from that used in Grosvenor et al. (2018) in that data are not filtered for the presence of sea ice, nor for *τ*_c_< 5 data points. Both data sets use the 3.7 μm *r*_e_ (*r*_e3.7_) for the *N*_d_ calculation.

**Figure 7:**
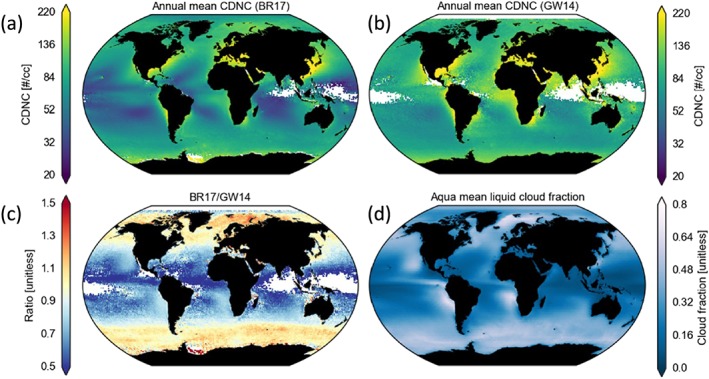
Annual mean N_d_ for (a) the BR17 data set and (b) the data set following GW14. (c) The ratio of N_d_ from BR17 to that from the data set following GW14. (d) Liquid cloud retrieval fraction from Aqua, averaged from 2003 to 2015 (note that the retrieval fraction is likely lower than the true liquid cloud fraction due to unsuccessful and unclassified retrievals).

In both data sets, *N*_d_ is highest near the continents, especially areas with high population density like Europe and the eastern coasts of North America and Asia, and lowest over the remote ocean regions (Figure 7). Annual means and seasonal cycles are most reliable for areas of large liquid cloud fraction; areas with lower liquid cloud fractions have much sparser, or even almost no, coverage in certain months or seasons.

The differences between the two data sets shown in Figure 7 are illustrative of how the necessary choices made in any *N*_d_ data set can influence results. Over the tropics and subtropics the BR17 values are smaller than those from GW14. One contributing factor to this observation is the use of an 80% cloud fraction screening in the latter data set. This screening for higher cloud fraction may lead to a sampling bias, since cloud fraction and *N*_d_ are correlated (Gryspeerdt et al., 2016). On the other hand, *r*_e_ biases are expected to be reduced at high cloud fractions (Wood & Hartmann, 2006). Since MODIS *r*_e_ tends to be positively biased relative to other *r*_e_ observations the expectation is that *r*_e_ values would be reduced at higher cloud fractions and thus *N*_d_ would be higher. Additionally, the screening in BR17 that requires the 3.7‐μm channel *r*_e_ to be larger than the 2.1‐ and 1.6‐μm channel values can create a low bias when the *r*_e_ from the different channels have very similar values; for example, in stratocumulus cases where there is no drizzle and the cloud is relatively homogeneous (Painemal & Zuidema, 2011). In these cases, sensor noise, etc. can act to reorder the *r*_e_ sizes at random, and the screening will then produce a skewed distribution by keeping higher, and excluding lower, 3.7‐μm channel *r*_e_ values, ultimately biasing the *N*_d_ retrieval low. At high latitudes, the BR17 data set shows larger values than those from GW14. This is likely due to the lack of screening for SZA bias in BR17 beyond what is done in the operational MODIS Level 2 cloud product, which was shown in GW14 to lead to large overestimates in *N*_d_ for SZA > 65° (see section 2.4.5).

**Figure 8:**
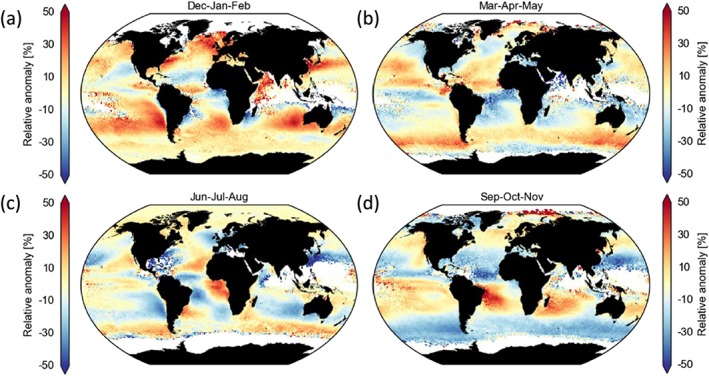
Relative anomalies of N_d_ for (a) December‐January‐February, (b) March‐April‐May, (c) June‐July‐August, and (d) September‐October‐November from the BR17 data set.

Now we break down the data sets by seasonal relative anomalies in Figure 8 for just the BR17 data set. In both the BR17 and GW14 (not shown) data sets, the large *N*_d_ values observed off the eastern coasts of North America and Asia peak in boreal winter. This is not consistent with what is expected from column Aerosol Optical Depth (AOD) retrieved by MODIS/Aqua or the seasonality of anthropogenic sulfate transport from chemical transport models, which instead suggests a peak in spring or summer (Berg et al., 2008; Luan & Jaeglé, 2013). However, additional independent evidence that there is an aerosol indirect effect peaking in winter off the east coast of China comes from both modeling studies and independent observations (Bennartz et al., 2011; Berg et al., 2008), although the mechanism that causes the effect to peak in winter is not fully understood. One possibility is that capping inversions caused by cold air outbreaks are more prevalent in winter, which act to contain and concentrate surface emitted pollution. This is supported by the higher number of days in winter compared to summer with low‐altitude areal cloud fractions that are greater than 80% (for which *N*_d_ retrievals are attempted) in the GW14 data set, indicating the presence of stratocumulus clouds and capping inversions. It is also possible that *N*_d_ is actually high in summer, but the MODIS‐derived *N*_d_ are biased low due to the prevalence of cumulus clouds for which *N*_d_ retrievals are more problematic.

**Figure 9:**
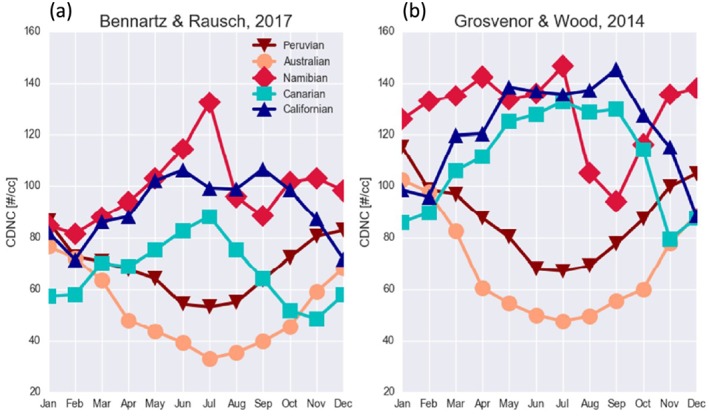
Average values of N_d_ from 2003 to 2015 for the five subtropical stratocumulus regions identified in Klein and Hartmann (1993) for (a) the BR17 data set and (b) the GW14 data set. Northern Hemisphere decks are shown in shades of blue and Southern Hemisphere decks in shades of red.

Over most of the subtropical regions dominated by marine stratocumulus, there is a clear seasonal cycle that peaks in local summer and troughs in local winter. This can be seen more clearly in Figure 9, which shows the seasonal cycle based on monthly averages for the five subtropical stratocumulus regions identified in Klein and Hartmann (1993). Both data sets show the same seasonal pattern, although the GW14 data set shows generally higher values, which is consistent with the global maps shown earlier. The southeast Atlantic (Namibian stratocumulus deck) stands out as an exception, with nearly the reverse seasonality of the other four subtropical stratocumulus regions. The *N*_d_ peak in July for the southeast Atlantic, along with enhanced September, October, November (SON) *N*_d_ values near Madagascar, is consistent with the progression of the biomass burning season on the African continent (BR17). However, the relatively low SON *N*_d_ values over the southeast Atlantic pose a challenge, as aerosol optical depth remains high over the region in September and October (Adebiyi & Zuidema, 2016).

### 3.2 N_d_ Satellite Intercomparison: MODIS and AATSR Instruments

**Figure 10:**
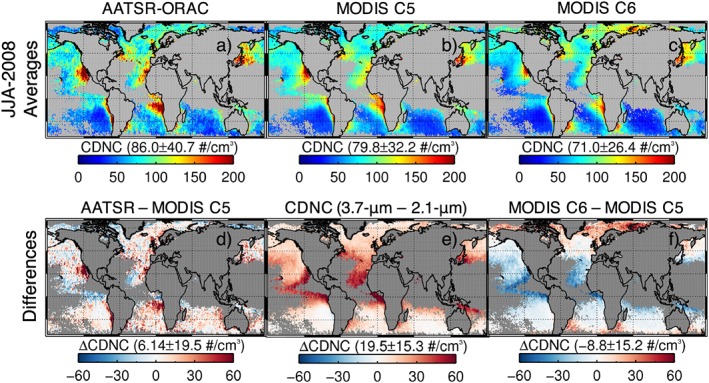
Mean N_d_ estimated using the 3.7‐μm r_e_ and τ_c_ for (a) Optimal Retrieval for Aerosol and Cloud (ORAC) retrieval applied to Advanced Along‐Track Scanning Radiometer (AATSR) observations, (b) the Collection 5.1 MODerate Imaging Spectroradiometer (MODIS) data (based on GW14), and (c) the Collection 6 MODIS data (based on BR17) using 3 months spanning June, July, and August 2008. Differences between (d) AATSR‐ORAC and MODIS Collection 5.1, (e) 3.7 and 2.1‐μm retrievals for MODIS Collection 5.1, and (f) MODIS Collection 6 and 5.1 differences are shown for the same period. N_d_ data are aggregated to daily temporal and 1×1° horizontal resolutions. Data from individual swaths at 1×1° resolution that has SZA > 65° and a cloud top height less than 3.2 km are excluded from the analysis for the C5 and AATSR data but are not excluded for the C6 data. Only pixels with more than 5 days of valid data for both instruments are shown. CDNC = cloud droplet number concentration.

The AATSR instrument is on board the ENVISAT satellite and observes at similar wavelength channels to MODIS and thus can be used as an alternative to MODIS for estimating *N*_d_ or to provide additional data. AATSR retrieves cloud properties using an optimal estimation framework in the Optimal Retrieval for Aerosol and Cloud (ORAC) algorithm (Poulsen et al., 2012). Since this is different to the approach used by MODIS, differences between the two *N*_d_ data sets are informative in terms of the uncertainty that is introduced by the retrieval methods. *N*_d_ from MODIS on Aqua (using both Collection 5.1 and Collection 6 data, hereafter C5 and C6, respectively) and AATSR are examined using 3 months of daytime observations during June, July, and August (JJA) of 2008 (Figure 10). The C5 data set is based upon GW14 as above except without the filtering for cloud fractions less than 80%. The C6 data set is BR17. See the figure caption for more details.

AATSR‐ORAC retrieves much larger *N*_d_ values in stratus‐dominated regions along the coast of Baja California, Chile, and Namibia by approximately 50%, while smaller values are generally found in the open ocean and at higher latitudes. On average the AATSR *N*_d_ retrieval is found to have a small positive bias with respect to the MODIS C5 product by approximately 7%. The large regional differences, particularly in stratocumulus‐dominated locations are primarily due to larger cloud optical depths (by approximately 5) in ORAC‐AATSR observations (not shown).

In agreement with the results found by Rausch et al. (2017), *N*_d_ tends to have lower values across the tropics and subtropics in C6 data compared to C5 (using the 3.7‐μm retrieved *r*_e_ in both cases). In Rausch et al. (2017) *N*_d_ differences were attributed to the corrections to the band‐averaged solar irradiances, atmospheric emission factors, and changes in cloud top pressure as used in the new C6 look up tables. On average, the root mean square differences among the three data sets examined are approximately within 20 cm^−3^ or ±20%.

These results indicate that uncertainties introduced by instrument errors and those from the retrieval algorithms are relatively small giving some confidence in the idea that they can be mostly neglected when averaging over large spatial scales. However, the retrievals are similar enough that they will all be subject to forward model errors such as those arising from cloud heterogeneity (see sections 2.3 and 2.4) in a similar manner and so the intercomparison cannot be used to draw conclusions about those types of errors, nor for those in the constants that are assumed for the *N*_d_ calculation since the same values were used in all of the retrievals.

Figure 10e shows that *N*_d_ retrieved from the 3.7‐μm channel (*N*_*d*3.7_) is about 20% larger than when using the 2.1‐μm channel (*N*_*d*2.1_), although in the stratocumulus regions the difference is much smaller. Since larger positive subpixel heterogeneity biases are expected for the 2.1‐μm *r*_e_ retrievals than for the 3.7 μm one (see section 2.4) the difference between *N*_d_ from the two channels is likely to give some indication of the severity of such effects and as a result an indication of the regions where the *N*_d_ retrievals might be most trusted. The low differences in the stratocumulus regions corroborate the expectation that the clouds in such regions are more homogeneous than in equatorial and midlatitude regions where more cumuliform clouds are expected. However, for stratocumulus it is also expected that *r*_e3.7_>*r*_e2.1_ due to the observation that *r*_e_ increases with height combined with the deeper penetration of the shorter wavelengths. Thus, in the absence of biases *N*_*d*2.1_>*N*_*d*3.7_ would be expected. It is possible that this penetration depth effect is canceled out by a more positive subpixel bias for *r*_e2.1_ resulting in a small overall *r*_e_ difference in stratocumulus.

The direction of the *N*_d_ difference (*N*_*d*3.7_>*N*_*d*2.1_) in regions outside the stratocumulus zones is consistent with that expected for positive biases in *r*_e2.1_ compared to *r*_e3.7_ due to heterogeneity. Many regions do not appear on these plots due to the filtering process applied; the heterogeneity of the filtered regions is likely to be even higher. However, as highlighted above, there are other factors that can affect the relative values of *r*_e2.1_ and *r*_e3.7_ and so the difference may not always be indicative of bias. Physical vertical variation of *r*_e_ may be different between stratocumulus and cumulus regions; for the latter suggestions of the presence of rain reversing the *r*_e3.7_>*r*_e2.1_ profile expected for stratocumulus have been made (Chang & Li, 2002; Nakajima et al., 2010a, 2010b; Suzuki et al., 2010), although Zhang et al. (2012) found that MODIS retrievals of *r*_e_ performed on model‐generated clouds were not significantly affected by the presence of precipitation. In section 2.4.4 it was also noted that the presence of a rain mode (i.e., a dual‐mode DSD), or the presence of a single cloud mode that is wider than that assumed by the MODIS retrieval (both of which could be argued as being more likely outside of stratocumulus regions) can cause opposing differences between *r*_e2.1_ and *r*_e3.7_.

It can also been seen that the regions of high *r*_e3.7_ minus *r*_e2.1_ values correlate well with the regions of high subpixel heterogeneity, as quantified using the *H*_*σ*_ parameter (Figure 3, see section 2.4.1), indicating that cloud heterogeneity is a potential cause of the *r*_e_ difference in agreement with Zhang et al. (2012). Overall, both Figures 3 and 10e indicate that the main stratocumulus regions and also the North Atlantic, North Pacific, and Southern Ocean regions are likely to exhibit the lowest *N*_d_ biases according to these metrics.

## 4 Newer Satellite Approaches

In this section we discuss newer satellite‐based methods that might potentially allow the production of superior *N*_d_ data sets than the more standard approach already discussed, particularly for challenging retrieval environments, or could inform and improve the existing methods.

### 4.1 Improvements for the MODIS‐Like Approach

Sections 2.3 and 2.6 listed numerous sources of uncertainty on *N*_d_ satellite estimates, several of which are related to an erroneous or simplistic representation of cloud layers in the forward model used for performing the retrievals. Recently, though, corrections or new retrieval methods have been developed to reduce these uncertainties. This section introduces potential solutions to errors on MODIS‐like retrievals due to (i) inconsistencies between vertically stratified cloud models, (ii) subpixel heterogeneities, and (iii) multilayer cloud conditions.

The model inconsistency uncertainty, which was discussed in section 2.3.1, refers to the fact that a vertically homogeneous profile of cloud properties (in particular *r*_e_) is assumed to retrieve *τ*_c_ and *r*_e_, while equation (11) assumes an adiabatic or subadiabatic profile of cloud parameters within the cloud layers. Two main issues arise from this approach. First, the *r*_e_ value used in equation (11) supposedly represents the value at cloud top, while Bennartz and Rausch (2017), Grosvenor et al. (2018), and Platnick (2000) demonstrated that it is representative of a value somewhat below cloud top. Second, inherent inconsistencies in the radiative transfer calculations occur, as *R*_vis_ and *R*_SWIR_ computed for two clouds with the same *r*_e_ at cloud top, but different vertical profiles, are different.

Grosvenor et al. (2018) suggest a parameterization of the first of the above‐mentioned errors as a function of *τ*_c_ only, which could potentially be used to correct this bias. However, this does not take into account the second issue listed above. The consequences of the second effect are not yet well studied in the literature. It should be mentioned that using a vertically homogeneous model for representing liquid clouds is a convenient approach for operational retrievals of *τ*_c_ and *r*_e_ as it does not require a high stratification of cloud layers and is therefore computationally efficient. Nevertheless, adiabatically stratified cloud models have already been developed and tested (Brenguier et al., 2000; Schüller et al., 2003, 2005) that allow *N*_d_ and the cloud thickness to be directly retrieved. These efforts have not yet been pursued for operational applications but may be a useful way to reduce the uncertainties of current *N*_d_ estimates.

Section 2.4.1 discussed the importance of subpixel variability, that is, the variability of cloud properties below the instrumental resolution, on cloud retrievals. Because this effect concerned the unresolved heterogeneity of cloud properties, it is difficult to directly correct through improvements of the retrieval methods. However, Zhang et al. (2016) predict the expected subpixel biases on *τ*_c_ and *r*_e_ based on a knowledge of the subpixel variability of *R*_vis_ and *R*_SWIR_. A Taylor expansion approach is used to compute the nonlinear relations between cloud properties and reflectances through derivative calculations and predict their subsequent effects on cloud retrievals. Zhang et al. (2016) demonstrated agreement between simulated and measured biases in MODIS retrievals, but only for one scene and using relatively coarse resolution (500 m) data to analyze the subpixel effects. Werner et al. (2018) extended the analysis using 30‐m resolution ASTER retrievals of *τ*_c_ and *r*_e_ to assess subpixel biases for 48 stratocumulus scenes and compared them to those predicted using the method of Zhang et al. (2016). Again, good agreement was found with correlation coefficients of 0.97 for the predicted *τ*_c_ bias and 0.8 for the *r*_e_ bias. This method thus seems adequate for correcting subpixel heterogeneity errors. A difficulty is that it requires knowledge of the variances and covariances of *R*_vis_ and *R*_SWIR_. While the subpixel heterogeneity over 1‐km regions for the *R*_vis_ channels (based on 250‐m resolution reterievals) is an operational product of MODIS C6, the corresponding value for *R*_SWIR_ is not. However, the results of Werner et al. (2018) also hint that such low‐resolution data may be reasonably adequate for this purpose.

Finally, section 2.4.6 mentioned the strong impact of multilayer conditions on liquid cloud retrievals when assuming a single cloud layer in the retrieval. A solution to this problem consists of allowing for a second cloud layer in the retrieval, and for example, applying variational methods (Rodgers, 2000). Sourdeval et al. (2014) demonstrated that combining visible, shortwave‐ and thermal‐infrared measurements from MODIS allows the retrieval of *τ*_c_ and *r*_e_ for a liquid layer, as well as the ice water path of an overlying ice cloud in the case of multilayer conditions. Sourdeval et al. (2016) later confirmed these theoretical conclusions by applying this multilayer retrieval method to measurements. Section 2.4.6 also discussed the strong potential impact of aerosols on cloud retrievals, which could also be corrected for by such multilayer retrieval methods. Waquet et al. (2009, 2013) used a variational method to retrieve properties of aerosol above liquid cloud layers, using the information contained in polarimetric and multiangular measurements. Such a scheme could be used to correct cloud retrievals by directly accounting for the presence of an aerosol layer in the retrieval algorithm.

### 4.2 N_d_ Retrievals Using Microwave LWP

Uncertainties in *N*_d_ stemming from biases in passive *τ*_c_ retrievals can be removed by casting *N*_d_ (equation (10)) in terms of the liquid water path and *r*_e_, if independent LWP retrievals are available. LWP can be retrieved by passive microwave sensors (several instruments are in space) with the advantage that these long wavelengths are not sensitive to aerosols. This *N*_d_ reformulation relies on the pseudo‐adiabatic relationship (Szczodrak et al., 2001):

(17) $$\text{LWP}=\frac{5}{9}\rho_{w}r_{e}\tau_{c}.$$

Substituting *τ*_c_ as a function of *L* in equation (10) yields

(18) $$N_{d}=\frac{6\sqrt{(}2)}{\mathrm{k\pi}\rho_{w}Q_{\text{ext}}^{3}}\frac{{\left(f_{\text{ad}}c_{w}\text{LWP}\right)}^{1/2}}{r_{e}^{3}}.$$

However, microwave retrievals of LWP could also include contributions from rain water path, which is undesirable since it is likely that the precipitating parts of the cloud do not obey the assumptions required for *N*_d_ retrievals. Ideally, only the cloud contribution to the water path would be used with the assumption that the rain region contributes little to the visible or SWIR reflectances and that the cloud region still obeys the *N*_d_ retrieval assumptions. Also, the undetected presence of larger rain drops would lead to LWP retrieval errors (Lebsock & Su, 2014). Retrievals from many of the microwave instruments attempt to return only the cloud part of the total water path, but the method used is fairly rudimentary since it is based on the assumption that contributions to the water path from rain only start to occur above a constant 180 g/m^2^ threshold (Hilburn & Wentz, 2008), whereas in reality this is likely to depend on the *N*_d_ value (i.e., droplet size; Seethala & Horváth, 2010). It may, therefore, be useful to consider additional screening for the presence of rain (see section 2.4.4) for these retrievals.

**Figure 11:**
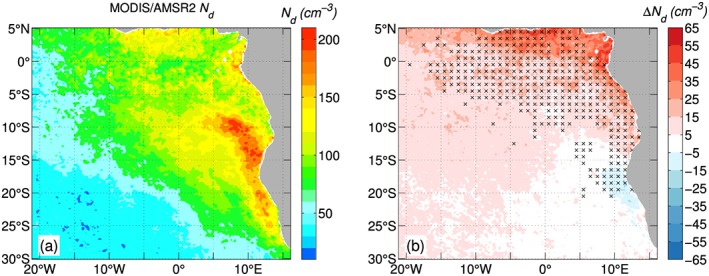
Satellite N_dMW_ for the period June to September 2016 for the southeast Atlantic region with liquid water path <250 g/m^2^. (a) Distribution of N_dMW_; (b) difference between N_dMW_ and MODerate Imaging Spectroradiometer (MODIS)‐only N_d_ derived from MODIS Clouds and Earth's Radiant Energy System (CERES) r_e_ (3.8‐μm channel) and τ_c_ (0.64‐μm channel). Crosses indicate MODIS June‐September level 3 aerosol optical depth higher than 0.2.

Figure 11 shows *N*_d,MW_ values calculated for overcast scenes from June to September 2016 using Advanced Microwave Scanning Radiometer 2 (AMSR2) LWP retrieved with the Wentz algorithm, version 7 (Wentz, 2013; Wentz & Meissner, 2000), and Aqua‐MODIS *r*_e_ (3.8 μm) estimated using the Clouds and Earth's Radiant Energy System (CERES) Edition‐4 algorithms (Minnis et al., 2010, 2011). Assumed constants are *k* = 0.8, *f*_ad_=1, and the *c*_w_ value was calculated using *T* = 283 K and *P* = 850 hPa. *N*_dMW_ follows a common pattern observed in subtropical marine boundary layer clouds, with high values along the coast, decreasing systematically to the west as the boundary layer deepens. The difference between *N*_dMW_ and *N*_d_ derived from MODIS‐CERES *r*_e_ and *τ*_c_ (0.64‐μm channel) is also depicted in Figure 11. On average, *N*_dMW_ is 10 cm^−3^ higher than its *τ*_c_−*r*_e_ counterpart. These differences are qualitatively consistent with the results of Bennartz and Harshvardhan (2007). Clouds near the coast at 20° S are very thin with LWP_MW_ smaller than MODIS LWP (Seethala & Horváth, 2010), which may help explain why *N*_dMW_ is less than *N*_d_. In contrast, some *N*_dMW_ > *N*_d_ values north of the equator could also be caused by precipitation and biases associated with the cloud temperature parameterization used in the Wentz algorithm (e.g., Seethala & Horváth, 2010) rather than absorbing aerosol.

As LWP_MW_ is insensitive to 3‐D radiative transfer (for nonprecipitating cloud scenes), spatial heterogeneity, and viewing geometry effects, *N*_dMW_ is less biased by these factors than MODIS‐only *N*_d_. For overcast stratiform clouds, LWP_MW_ is nearly unbiased relative to independent ground‐based microwave retrievals (Painemal et al., 2016). Thus, *N*_dMW_ is best suited for climatological studies of marine nonconvective clouds in cases where the standard visible/infrared *τ*_c_ is prone to biases due to overlying aerosols or spatial heterogeneity affecting the *τ*_c_ retrieval, although its difference from *N*_d_ estimates can also be used to motivate exploration of the physical processes and retrieval behavior. A strength is that areas where the spatial patterns of *N*_d_ and *N*_dMW_ agree well can be more robustly interpreted to reflect genuine aerosol activation within the cloudy boundary layer.

However, LWP_MW_ retrievals are less reliable for precipitating clouds with total water path greater than 180 g/m^2^ (Seethala & Horváth, 2010) and for broken cloudy scenes within the instrument field‐of‐view (Greenwald et al., 1997). In this regard, the microwave‐derived LWP estimates typically correspond to a spatial resolution of 12 × 7 km^2^ (footprint resolution for the 36.5‐GHz AMSR2 channel) or coarser, compared to the 1‐km^2^ resolution of MODIS *r*_e_ and *τ*_c_. For this reason, and because LWP_MW_ includes contributions from both the clear and cloud parts of the footprint, the cloud fraction within the footprint needs to also be considered (since N_d_ is only defined for cloudy regions), which may introduce some error due to uncertainty in cloud fraction observations. In addition, the coarse resolution of the LWP observations requires that the LWP for the cloudy part of the grid box is assumed to be uniformly distributed, which in reality it may not be. This method still relies on *r*_e_, to which it is even more sensitive than the method using *τ*_c_ and *r*_e_ (equation (11)) and thus is still subject to errors due to *r*_e_ biases discussed earlier. It is also possible to use equation (17) to remove the dependence of N_d_ upon *r*_e_; this method was explored by Bennartz (2007) and may be preferable in situations where *τ*_c_ retrievals are not affected by overlying aerosol layers.

### 4.3 Polarimetric Retrievals

Multidirectional polarization measurements provide an alternative method to infer *r*_e_ (Alexandrov et al., 2012; Bréon & Doutriaux‐Boucher, 2005; Bréon & Goloub, 1998). Compared to retrievals using total reflectance measurements in the shortwave infrared (section 2), polarimetric retrievals offer many advantages. In conditions when polarimetric retrievals are possible, they are minimally affected by vertical and horizontal cloud inhomogeneities (Alexandrov et al., 2012; Shang et al., 2015). Furthermore, polarimetry also allows the effective variance (*v*_e_) of the size distribution to be inferred. The ability to infer *v*_e_ for a DSD using polarimetry is of particular interest for retrievals of *N*_d_ since it allows an estimate of the *k* parameter (see section 2) using equation (13). In addition, by using Lorentz‐Mie theory and assuming a modified gamma distribution for the DSD, *n*(*r*), the mean particle extinction cross section can be calculated, since *n*(*r*) is fully described by the retrieved *r*_e_ and *v*_e_. However, in the presence of substantial subpixel inhomogeneity of *r*_e_ or multiple cloud layers with different droplet sizes assuming a modified gamma distribution for the DSD is not appropriate and a nonparametric size distribution retrieval method is required (Alexandrov et al., 2016). The maximum *v*_e_ allowed for a gamma distribution is 0.5 and so any form of heterogeneity that has *v*_e_> 0.5 cannot be captured.

For a detailed description and evaluation of the polarimetry retrievals of *r*_e_ and *v*_e_, we refer to Alexandrov et al. (2012, 2016). In short, the polarimetry method uses the primary and secondary cloudbow structures that appear in the polarized reflectances at scattering angles between about 135 and 165° . The polarization signal is determined by light undergoing low orders of scattering and the retrievals therefore pertain to roughly the first optical depth into the top of the cloud. The structure of the cloudbow (location of zero crossings, minima and maxima) depends uniquely on *n*(*r*) and can be accurately calculated using Lorentz‐Mie theory. By focusing on fitting the locations and relative strengths of the cloudbow features, sensitivity to issues that affect the absolute value of the reflectances, such as cloud fraction, 3‐D radiative transfer, mixed‐phase conditions, and overlying cirrus, can be minimized (Alexandrov et al., 2012). Furthermore, the presence of a cloudbow structure in the measurements provides a virtually unambiguous detection of liquid drops, and thus cloud phase (Goloub et al., 2000; van Diedenhoven et al., 2012). Polarimetric retrievals of *r*_e_ and *v*_e_ require multidirectional polarized reflectance measurements. The measurements need to be of sufficient angular resolution for the features to be sampled, although the specific requirements for angular resolution and sampling have not been well quantified yet. In addition, scattering angles between 135 and 165° need to be sampled and this sampling is determined by the SZA, RAZ, and sampled viewing angles, and thus, for a satellite instrument, varies as a function of latitude and time of year.

To date, space‐based multidirectional polarization measurements have only been provided by the three POLDER instruments. Most notably, the POLDER instrument on the PARASOL platform was part of the A‐Train constellation between December 2004 and December 2009, allowing the combination of its measurements with MODIS, CALIPSO, CLOUDSAT, and other instruments. Zeng et al. (2014) made use of this by combining *r*_e_ from POLDER with the layer‐integrated depolarization ratio measurements from CALIPSO in order to retrieve cloud top *N*_d_; see section 4.4 for more details. The operational POLDER retrieval algorithm aggregates measurements from 150 × 150 km^2^ to compensate for the limited angular sampling. At such large spatial scales, subpixel inhomogeneity of *r*_e_ can be substantial, potentially leading to biases in the retrieved DSDs and derived *N*_d_ (Miller et al., 2017; Zeng et al., 2014). However, results from Shang et al. (2015) suggest that areas for data aggregation can be reduced by about a factor of 3. Another consideration for polarimetric retrievals is that the *r*_e_ is representative of that very close to cloud top (within approximately one optical depth) and so is potentially prone to evaporation effects related to entrainment, which have the potential to reduce *r*_e_. However, it is suggested that extreme inhomogeneous mixing occurs at the top of stratocumulus, which does not change *r*_e_ (see also section 4.5, Brenguier et al., 2011; Burnet & Brenguier, 2007). This is consistent with the in situ stratocumulus study of Painemal and Zuidema (2011) that did not show an *r*_e_ decrease at cloud top despite a reduction in the liquid water content. Sampling restrictions to low *r*_e_ variability regions are also required for POLDER retrievals, which may lead to sampling biases relative to less restrictive retrievals (Bréon & Doutriaux‐Boucher, 2005; Rosenfeld & Feingold, 2003).

**Figure 12:**
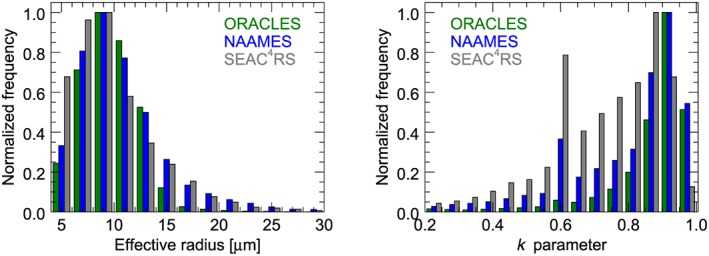
Histograms of cloud top r_e_ and k retrieved by the Research Scanning Polarimeter during the 2016 ORACLES (Marine stratus off Namibian coast), 2015/2016 NAAMES (Marine stratocumulus over North Atlantic), and 2013 SEAC4RS (Cumulus congestus over southern United States and gulf of Mexico) campaigns.

In addition to POLDER, various airborne polarimeters have been deployed with spatial resolutions on the order of 10–100 m. Most notably, the RSP provides a high angular resolution (about 0.8° ) that allows operational retrievals of *r*_e_ and *v*_e_ for every footprint with the required scattering angle range (Alexandrov et al., 2015, 2016). RSP statistics of *r*_e_ and *k* (calculated from *v*_e_ using equation (13)) for liquid‐containing clouds observed during various campaigns are given in Figure 12.

Future opportunities for space‐based multiangle polarimetry for cloud retrievals include the 3MI instruments on ESA's METOP series that are based on POLDER (Marbach et al., 2013), as well as instruments on the Ukrainian Aerosol‐UA satellite (Milinevsky et al., 2016). Furthermore, the Hyper Angular Rainbow Polarimeter (HARP) is a cube sat mission due to be launched in 2018 that will provide data for selected targets during its 3‐month lifetime (Martins, 2016). In addition, a version of HARP is planned to be on the NASA Plankton, Aerosol, Clouds, Ocean Ecosystem (PACE) mission to be launched after 2022. HARP will have a spatial sampling of about 4 × 4 km^2^ and an angular sampling sufficient to retrieve DSDs on a pixel level.

### 4.4 Active Spaceborne Instruments and Multi‐Instrument Retrievals

Some of the issues related to the *N*_d_ retrieval as described in section 2 may be alleviated by incorporating the information from multiple instruments, including active instruments (i.e., those that emit a laser, e.g., lidar, or radar beam and use the return signal to determine cloud properties). With the launch of a cloud radar on CloudSat (Stephens et al., 2002) and a cloud‐aerosol lidar on CALIPSO, multi‐instrument retrieval capabilities have been enhanced to also incorporate vertically resolved information. However, current retrieval approaches generally also require *r*_e_ observations from passive instruments. Combining observations with different viewing geometries, overpass times, and spatial and temporal resolutions can be problematic. The assumptions made in each instrument's retrieval may also be difficult to reconcile if the retrieved physical products from each instrument are used in the multi‐instrument retrieval (Chen et al., 2011; McCoy et al., 2014).

Early work toward a dedicated *N*_d_ retrieval from active remote sensing by Austin and Stephens (2001) used an optimal estimation scheme, combining profiles of cloud radar reflectivity factors and *τ*_c_ provided by radiometric retrievals. Further development of this product, notably regarding ice clouds, led to the CloudSat CWC product. Mace et al. (2016) developed a Bayesian optimal estimation approach to combine vertical profiles of radar reflectivities from CloudSat with solar reflectances from MODIS to characterize cloud and precipitation properties.

Hu et al. (2007) described a technique to determine *N*_d_ multiplied by the *k* parameter, denoted as *N*_*e*_, for a layer near cloud top by using the layer‐integrated depolarization ratio from Cloud‐Aerosol Lidar with Orthogonal Polarization (CALIOP) combined with a retrieval of *r*_e_ from MODIS. Their approach has the advantage that it does not require adiabaticity assumptions, although there is some possibility for errors caused by vertical mismatches between the depolarization ratio and the *r*_e_ measurement that may depend upon cloud conditions. The liquid water content over the sampled cloud layer can also be obtained with this technique.

Zeng et al. (2014) showed global comparisons of these CALIOP‐based retrievals of *N*_*e*_ that used either MODIS or POLDER *r*_e_ and of MODIS‐only retrievals (using *τ*_c_ and *r*_e_ as in equation (11)) and POLDER‐only retrievals (using the same technique as for MODIS, but with POLDER derived *τ*_c_ and *r*_e_). The use of POLDER *r*_e_ may help to circumvent some of the issues related to *r*_e_ from MODIS that were described in section 2.4. For the MODIS retrievals they used *r*_e3.7_, assumed *f*_ad_=0.8 and restricted the analysis to pixels with *τ*_c_>5. Since it is likely that *f*_ad_=0.8 is an overestimate of the true value (e.g., Painemal & Zuidema, 2011, observed a mean value of 0.7 for the VOCALS observations in SE Pacific stratocumulus) and it is also possible that the near cloud top *N*_*e*_ retrieved from CALIOP may be lower than that deeper into the cloud due to entrainment related evaporation, the expectation is that the MODIS‐only *N*_*e*_ would be higher than the CALIOP‐MODIS *N*_*e*_. However, other factors could cause differences too such as the different dependencies upon *r*_e_ of the CALIOP‐based and MODIS‐only retrievals.

The results confirmed the expected result of a larger MODIS‐only *N*_*e*_ compared to that from CALIOP‐MODIS but with fairly small relative differences of close to 20% in many regions of the globe. The regions where this was not the case were the equatorial region and the subtropical trade cumulus regions, which might be expected to have lower *f*_ad_ values and to suffer more from cloud top entrainment. The lack of variation in the difference between the two retrievals is actually quite striking, suggesting that spatial variability of the time‐mean *f*_ad_, cloud top entrainment, or the other factors mentioned above is not particularly large across many regions.

Time series revealed that for large parts of the year the CALIOP‐MODIS *N*_*e*_ was larger than that of MODIS‐only for the SE Pacific, Californian, and SE Atlantic stratocumulus regions, which may have been indicative of an *f*_ad_>0.8 and/or less cloud top entrainment for those times. The spatial pattern of the *N*_*e*_ differences between the two retrievals was similar to that of the differences between *r*_e2.1_ and *r*_e3.7_, and there was correlation (*r* = 0.53) between them. This indicates that cloud heterogeneity was playing a role in creating differences between the MODIS‐only and CALIOP‐MODIS *N*_*e*_, either through increasing *r*_e_ or *τ*_c_ retrieval errors, or through a correlation with reduced cloud adiabaticity, and/or enhanced entrainment. Higher *r*_e3.7_ values were also associated with larger differences, potentially implicating the production of drizzle in a similar manner. The use of POLDER *r*_e_ instead of MODIS *r*_e_ in the CALIOP algorithm led to much higher *N*_*e*_ values; for example, annual averages of around 600 cm^−3^ were found near the coast in the SE Pacific. Since the true *N*_d_ is given by *N*_*e*_ divided by *k* the actual *N*_d_ is likely to be even higher. Such values are significantly higher than those suggested by the VOCALS field campaign measurements, which showed 90th percentile values up to 450 cm^−3^ in that region and median values of around 225 cm^−3^ or less (Bretherton et al., 2010). These aircraft measurements are more consistent with those calculated using the MODIS *r*_e_ in combination with CALIOP. This indicates that the POLDER *r*_e_ may be affected by cloud top entrainment resulting in an underestimate of the *r*_e_ in the layer sampled by CALIOP and an overestimate of *N*_*e*_. An alternative hypothesis is that the sampling restrictions necessitated for POLDER retrievals (i.e., low *r*_e_ variability) preferentially sample low *r*_e_ regions, which are likely to have high *N*_d_; in this case the VOCALS measurements may not be representative.

It would be useful to get to the bottom of such issues since the combination of POLDER *r*_e_ and CALIOP as described in Zeng et al. (2014) is potentially very promising for *N*_d_ retrievals given the fact that POLDER *r*_e_ is likely to be less affected by cloud heterogeneity compared to MODIS and the use of CALIOP sidesteps the issue of assessing the degree of cloud adiabaticity. In addition, POLDER allows a direct retrieval of the *k* parameter, which is also required to obtain the true *N*_d_ value from *N*_*e*_. However, the issues related to changes in *r*_e_ and *N*_d_ near cloud top due to entrainment and other processes must first be resolved; the use of LES‐generated cloud fields may prove useful in this respect.

### 4.5 N_d_ Retrievals for Convective Clouds

Retrieving *N*_d_ from convective clouds is especially challenging (see section 2). However, satellite imagers with a high resolution in the channels that allow the retrieval of cloud top temperature (*T*_top_) and *r*_e_ at the scale of small convective towers (e.g., the 375‐m resolution of the Suomi/NPP satellite instrument, Rosenfeld, Liu, et al., 2014) make such retrievals feasible (Rosenfeld, Fischman, et al., 2014; Rosenfeld et al., 2016). Aircraft observations in convective clouds have shown that *r*_e_ as a function of height above cloud base, *r*_e_(*z*), is close to that expected from adiabatic clouds (Freud et al., 2011; Pawlowska et al., 2000). Burnet and Brenguier (2007) explained this as being a result of the nearly inhomogeneous nature of cloud evaporation through entrainment, whereby droplets bordering the entraining air evaporate completely. After complete mixing the remaining cloud drops only evaporate to a small extent, thus preserving *r*_e_ as nearly adiabatic but reducing the cloud liquid water content and *N*_d_. Beals et al. (2015) demonstrated this process by measuring the millimeter‐scale microstructure of convective clouds. However, this is only true for the portion of the convective towers that do not experience significant collision coalescence, since this reduces *N*_d_ and increases *r*_e_ relative to that expected from the adiabatic assumption. Rosenfeld, Fischman, et al. (2014) suggest that insignificant collision coalescence occurs for $r_{e}\;≲$ 14 μm and thus restrict retrievals to clouds where this is the case.

The assumption of an adiabatic *r*_e_ profile allows the observed *r*_e_ of a developing cloud top at a given height, along with *T*_top_ and estimates of cloud base temperature (*T*_base_) and cloud base pressure (*P*_base_), to be used to estimate the adiabatic (or cloud base) *N*_d_ (*N*_d,ad_). The theoretical adiabatic liquid water content (*L*_*a*_) can be calculated for the observed *T*_top_ using the estimated *T*_base_ and *P*_base_. Rosenfeld, Fischman, et al. (2014) and Rosenfeld et al. (2016) employed the method of Zhu et al. (2014) who approximated *T*_base_ using the warmest cloudy pixels in a field of convective clouds and derived an accuracy of ±1^∘^C. *P*_base_ was estimated from *T*_base_ using reanalysis data. A constant *k* value of 0.79 (Freud et al., 2011) is assumed in order to calculate the volume radius (*r*_v_) from the observed *r*_e_ using equation (7). *N*_d,ad_ can then be obtained by dividing the *L*_*a*_ by the mass of a droplet that has this estimated *r*_v_ value following equation (8). The method can also be refined to take into account the degree of adiabaticity of the cloud (Freud et al., 2011) in order to estimate the actual *N*_d_ at the observation height. The accuracy of the *N*_d_ retrieval for deep convection is increased by sampling clouds within various stages of vertical development.

Rosenfeld, Fischman, et al. (2014) and Rosenfeld et al. (2016) also use the retrievals of *N*_d,ad_ to estimate a cloud base CCN concentration via an estimate of the cloud base updraft. The latter was found to be linearly correlated to cloud base height in Zheng and Rosenfeld (2015), which is in turn obtained from *T*_base_ and reanalysis data. CCN retrievals using this method were compared against surface‐based measurements of CCN at four sites (∼40 data points) in Rosenfeld et al. (2016); a mean underestimate of 14 ± 30% was found with a correlation coefficient of 0.76. Since the 30% variability of the bias combines the errors of *N*_d,ad_, cloud base updraft and ground‐based CCN measurements, this suggests that the accuracy of the retrieved *N*_d,ad_ alone is likely better than 30%.

### 4.6 N_d_ Retrievals Based on Reanalysis Models

Other less direct ways to estimate *N*_d_ via satellite data have been presented in the literature, for example, the use of an aerosol reanalysis model (McCoy et al., 2017, 2018), which incorporates aerosol optical depth information from satellite along with an aerosol emission and transport model. The latter study showed that sulfate aerosol mass mixing ratio from the MERRA2 reanalysis could be used to predict the GW14 *N*_d_ data set (Grosvenor & Wood, 2014; see section 3) derived using MODIS data. This result was supported by analysis of decadal trends in OMI‐observed SO2, MERRA2 SO4, and GW14 Nd (McCoy et al., 2018). The advantage of reanalysis data is that it will not be affected by cloud heterogeneity issues and so may prove useful in conditions where retrievals prove difficult, such as in cumulus clouds. The disadvantage is that it relies on uncertain model processes (e.g., precipitation scavenging of aerosol), and it is unlikely to represent physical cloud processes that affect *N*_d_ beyond activation at cloud base. In addition, it is unclear how well the relationships between sulfate and *N*_d_ that were developed in the regions where GW14 provided *N*_d_ retrievals (high cloud fraction, liquid‐topped low cloud, etc) extend to other regions.

## 5 Ground‐Based Remote Sensing Approaches

In this section we turn to ground‐based instruments, as opposed to those on satellites, which have been the focus of the discussion so far. *N*_d_ data from ground‐based instruments can complement that from satellite and may also be useful for estimating uncertainties in both types of measurement through data set intercomparisons since the two approaches use quite different techniques. Ground‐based instruments are observing clouds from below instead of above. This means that instruments that rely on light from the Sun observe transmitted rather than reflected light and that the beams from active instrument first encounter cloud base rather than cloud top, which is likely to be especially important for wavelengths that do not penetrate far into cloud (e.g., cloud lidar). However, when combined with certain cloud radars that are sensitive to the liquid water drops at cloud top there is a sampling of both the cloud base and cloud top. Similar active instruments in space would only observe the cloud top. Another important difference is that ground‐based observations are likely to be performed at much higher spatial and temporal resolutions, although generally only at one location, giving less spatial coverage than with a satellite.

Active and passive remote sensing at optical or microwave frequencies (e.g., by means of lidar and radar measurements) have proven to be suitable techniques for the determination of cloud microphysical properties. The underlying physical principles that are utilized to derive information about clouds can be summarized as follows:

1. *Geometrical scattering at optical wavelengths.* As was already pointed out in equation (2) and the corresponding text, cloud droplets can be considered as geometric scatterers at optical wavelengths, making the extinction coefficient, which can be measured with lidar, a function of solely the number size distribution of the cloud droplets.
2. *Rayleigh scattering at microwave wavelengths.* In the microwave regime, as is the case for Ka and W‐band cloud radars, cloud droplets are much smaller than the wavelength of the radiation, allowing the application of Rayleigh scattering theory. In such a case the reflectivity factor, *Z*, received with cloud radar is a function of the sixth moment of the droplet number size distribution (*n*(*r*)):
  (19) $$Z\approx 2^{6}\;\int_{0}^{\infty }n(r)\;r^{6}\;dr$$
3. *Multiple scattering.* Even though the overall extinction efficiency approaches a value of 2 in the optical wavelength regime, the angular distribution of the light scattering is a strong function of the effective size of the cloud droplets. With increasing droplet size, an increasing amount of light is scattered into the forward direction. The fraction of forward‐scattered light that remains in the field of view of the lidar can still be involved in subsequent backscattering processes. The backscattering signal produced by these multiply scattered photons adds an additional contribution to the received single‐scattering signal. In addition, also the polarization state of the multiply‐scattered light is different to the polarization state of light retuned after a single‐scattering process. Using appropriate lidar techniques, the intensity of the multiple scattering and its effects on the polarization state of the returned light can be observed and related to the DSD.
4. *Passive radiation measurements.* The aforementioned range‐resolved observations of scattering by cloud droplets benefit strongly from additional availability of column‐integrated measurements of the scattering properties at optical, infrared, and microwave wavelengths. For instance, the absorption of microwave radiation by liquid water droplets is a function of their mass concentration and thus of the liquid water path. It can thus be used to constrain retrievals of profiles of cloud microphysical properties.

In the course of this section, techniques that rely on one or several of the above‐mentioned physical principles are introduced.

Basic methods to obtain the droplet concentration from ground‐based active and passive remote sensing observations are presented in the literature (Boers et al., 2000; Brandau et al., 2010; Dong & Mace, 2003; Frisch et al., 2002, 1998; Mace & Sassen, 2000; Martucci & O'Dowd, 2011; Sassen et al., 1999). These methods use either profiles of the cloud radar reflectivity factor (*Z*) or the lidar extinction in combination with observations from different passive instruments, for example, the liquid water path (LWP) obtained from a microwave radiometer.

**Figure 13:**
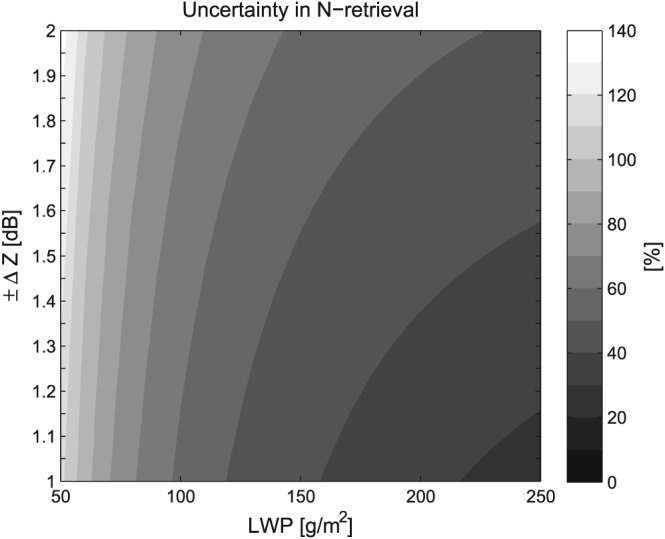
N_d_ uncertainty as a function of liquid water path (LWP) and observational errors in Z (radar reflectivity factor, see equation (19) for a LWP uncertainty of ±30 g/m^3^.

Usually, a mono‐modal DSD function, like gamma or log normal, is assumed to compute the moments of the DSD and, along with the constraint of the observed LWP, to link the radar reflectivity factor to the cloud liquid water content (*L*) . The use of a constant ratio between the cubes of the volume mean radius of the DSD and the effective radius (equation (7)) allows for an estimate of *N*_d_ from the *Z*‐*L* relationship. The uncertainties associated with such a retrieval of *N*_d_ are mainly related to the assumptions of the DSD and to the presence of observational errors. For example, theoretical error estimates show (Figure 13) that an LWP uncertainty of ±30 g/m^2^ and cloud radar calibration errors between ±1 and ±2 dB lead to relative retrieval errors in *N*_d_ of up to 140% and are large especially for clouds with low LWP (Knist, 2014). The accuracy of *N*_d_ retrievals is also limited by the assumptions relating to the DSD and the uncertainty of the *k* coefficient obtained from in situ data (Brandau et al., 2010; Miles et al., 2000). Knist (2014) shows that the use of the value of *k* = 0.74 ± 0.061 (Brenguier et al., 2011) leads to an *N*_d_ retrieval error of around 20%. This is higher than expected for the MODIS retrieval where equation (16) suggests only an 8% error. This is because ground radar‐based techniques use the relationship between *N*_d_ and higher‐order moments compared to those used for the MODIS retrieval and so are more sensitive to *k*. So, while with ground observations cloud properties can be retrieved with great spatial and temporal detail, stringent requirements need to be placed upon the accuracy of the observations (Knist, 2014) in order to reach below a sufficient level of uncertainty and serve as useful references for the validation of space‐based *N*_d_ retrievals.

Rémillard et al. (2013) proposed a retrieval technique that is based on the combination of radar and radiometer measurements, with additional constraints being provided by a cloud condensational growth model with a lognormal cloud DSD to describe the *Z* profile. Rémillard et al. (2013) demonstrated that the vertical gradient of *Z* combined with steady state supersaturation estimates from the radar mean Doppler velocity can be used to constrain the relationship between *N*_d_ and *k*. Subsequently, assuming that *k* is constant with height, they allow for *N*_d_ to vary with height. The proposed method reduces the uncertainty in the estimation of *r*_e_ and column‐averaged *k*. However, one limitation of the proposed technique is that it requires nonprecipitating conditions or that at least drizzle does not dominate the *Z* profile.

In contrast to methods that combine radar and passive microwave observations, Fielding et al. (2014, 2015) proposed the ENsemble ClOud REtrieval (ENCORE) method and retrieved column‐mean *N*_d_ using measurements from cloud radar, lidar, and shortwave spectral radiometers. ENCORE firstly enables a 3‐D retrieval by an iterative Ensemble Kalman Filter approach, in which the ensemble generates error statistics. Second, ENCORE fully accounts for 3‐D radiative effects in the retrieval by including 3‐D radiative transfer as a forward model. This is particularly important for retrieving cumulus cloud properties that are highly heterogeneous. Third, the use of shortwave radiation not only provides a direct constraint in cloud optical properties but also alleviates the issue that microwave radiometers have a much larger field‐of‐view compared to radar/lidar. No assumption for the *L* profile is needed for nonprecipitating clouds.

Promising techniques for the determination of cloud microphysical properties that solely rely on measurements in the optical wavelength regime were reported by Schmidt et al. (2013) and Donovan et al. (2015). Both approaches are based on the relationship between *r*_e_ and the intensity of forward scattering. Based on a forward iterative algorithm that uses the measured signals from a Raman lidar at two fields of view Schmidt et al. (2013), Schmidt et al. (2014) demonstrated the derivation of profiles of the *r*_e_, extinction coefficient, *L*, and *N*_d_ of liquid water clouds. The uniqueness of this dual‐field‐of‐view method is that light is detected which was scattered in the forward direction by cloud droplets but backscattered inelastically by nitrogen molecules. The scattering phase function of inelastic Raman scattering by nitrogen molecules is only dependent on the known concentration of the nitrogen molecules (which is a function of pressure and temperature). Any effects of a droplet‐size‐dependent backscattering intensity of the multiply‐scattered light are thereby eliminated, which makes the technique superior to previously published multiple‐scattering retrievals (such as of Bissonnette et al., 2007). The actual retrieval of *N*_d_ used by Schmidt et al. (2014, 2015) was based on the scaling of a modified gamma distribution toward the retrieved profiles of *r*_e_ and *L* following the approach of Brenguier et al. (2000). A *k* value of 0.75 was chosen based on Lu and Seinfeld (2006), and the uncertainties in the retrieved *N*_d_ values were reported to be in the range of 25–75% (Schmidt et al., 2014).

In addition to its effect on the width of the forward‐scattering peak, multiple scattering of light by spherical cloud droplets causes an observable modification of the polarization state of the backscattered light (Bissonnette, 2005). Single‐scattered light does not change its polarization state when scattered exactly into the backward direction of 180°. Multiple scattering, however, involves scattering angles different from 180°. Thus, the profile of the polarization state of the light returned from a cloud is related to the intensity of multiple scattering which is again a function of particle size and concentration. Donovan et al. (2015) exploit this principle to use lidar‐based depolarization measurements to derive profiles of *r*_e_ and *L* as well as column‐mean values of *N*_d_ and the liquid water lapse rate, from which the subadiabatic factor *f*_a*d*_ (equation (9)) can be calculated. To retrieve this list of parameters, an optimal estimation scheme in combination with lookup tables created with a Monte Carlo multiple scattering model was utilized. Similar to the works of Schmidt et al. (2013, 2014) the approach of Donovan et al. (2015) also uses a modified gamma distribution (with *k* = 0.75) to describe *n*(*r*). The uncertainty of retrieved *N*_d_ reported by Donovan et al. (2015) was between 25% and 50%.

It is noteworthy that both authors of the lidar‐based studies highlight the observed relationship between *N*_d_ derived at cloud base and aerosol particle number concentration derived below cloud base. Using vertical‐velocity observations of a colocated Doppler lidar, Schmidt et al. (2014) in addition showed that clouds are more adiabatic and more susceptible to the aerosol conditions at cloud base in updraft regions compared to downdraft regions.

Drawbacks of the lidar‐based retrievals of *N*_d_ are the inability to penetrate optical depths larger than approximately 3. Nevertheless, this limitation makes the techniques still comparable to the MODIS‐based retrievals which (for *r*_e_, although not for *τ*_c_) are also limited to the first 2–3 optical depths below cloud top, albeit that lidar ground‐based systems are usually observing the cloud base.

Radar‐based remote sensing methods using mono‐modal DSDs produce biased results when drizzle‐sized particles are present in the scattering volume. Drizzle droplets contribute substantially to the radar reflectivity factor, while their contribution to *N*_d_ and *L* is rather small (Krasnov & Russchenberg, 2006). Usage of instrument synergies and new data evaluation techniques may allow drizzle‐contaminated cloud regions to be identified in order to disregard them when using approaches that require a mono‐modal DSD. Acquistapace et al. (2016) demonstrate the applicability of higher‐order moments such as the skewness of the cloud radar Doppler spectrum for the identification of drizzling and drizzle‐producing regions in a cloud. Based on combined observations of lidar, cloud radar, and microwave radiometer, drizzle‐containing regions in a cloud are also identified within the widely applied Cloudnet retrieval (Illingworth et al., 2007).

## 6 Conclusions and Recommendations

Cloud droplet number concentration, *N*_d_, is a key parameter for understanding cloud processes. It is of particular interest for investigations of aerosol‐cloud interactions. Since no standard satellite retrieval for *N*_d_ exists, this quantity is currently inferred from standard passive visible and shortwave infrared retrievals of cloud optical depth, *τ*_c_, cloud droplet effective radius, *r*_e_, and cloud temperature. In order to derive *N*_d_, certain assumptions are commonly made: (i) *N*_d_ is constant with height in a cloud and (ii) cloud liquid water content increases monotonically at a constant fraction of its adiabatic value. Aircraft data and cloud‐resolving simulations tend to confirm that assumption (i) is frequently fulfilled. As for assumption (ii), different observations, especially from ground‐based remote sensing, suggest that cloud water profiles are substantially subadiabatic, with a subadiabatic factor, *f*_ad_, of about 0.66 ± 0.22.

Further uncertainties are introduced by the assumption that the volume‐mean cloud droplet radius, *r*_v_, scales with *r*_e_. According to aircraft observations, the scaling parameter *k* has a value of 0.80 ± 0.13. Finally, satellite retrievals of *τ*_c_ and *r*_e_ are uncertain, particularly due to violations of the assumptions of plane‐parallel homogeneous clouds and of negligible net horizontal photon transport across pixel boundaries (the independent pixel approximation in 1‐D radiative transfer), that are made for standard retrievals. The combination of all of these uncertainties leads to an overall uncertainty of around 78% for the *N*_d_ estimates at the pixel level for the “best case scenario” of relatively homogeneous stratocumulus clouds with a SZA < 65°, viewing angle < 55° and *τ*_c_> 5. If these conditions are not met then the *N*_d_ estimate is even more uncertain, for example at higher SZAs, for situations in which the liquid water cloud for which *N*_d_ is to be estimated is obscured by (semi‐)transparent overlying clouds/aerosol layers or for cumuliform clouds.

However, it is likely that uncertainties in the area averaged (here 1×1° regions were considered) *N*_d_ would be lower than the pixel‐level uncertainties through the cancelation of random errors. It is currently unknown which of the errors are random; here it is argued that this is likely to be the case for instrumental uncertainties but not for parameters such as *f*_ad_ and *k*, nor for errors in *τ*_c_ and *r*_e_ arising from cloud heterogeneity, due to the likelihood of cloud conditions being similar over 1° ×1° regions. Assuming the instrumental errors are random reduces the uncertainty to around 50%. Since it is possible that some of the other uncertainties are also random then this is likely to be an overestimate. This large difference in the uncertainty estimate highlights the need for a better characterization of whether such errors are random or not.

The few existing evaluation studies using reference observations from aircraft suggest, however, a negligible bias for the satellite‐derived *N*_d_ with little variability of the bias. The discrepancy with the uncertainty estimate above likely arises due to the presence of systematic offset biases that lead to little variability in the bias but potentially give large absolute errors, in addition to the fact that the comparisons were made for optimal retrieval conditions. Indeed, it has been shown that the low bias compared to aircraft was the result of compensating errors. This suggests that *N*_d_ retrieval accuracy could be significantly improved if such systematic biases could be characterized, which would likely need more comparisons to aircraft profiles. Currently, these have only been performed for specific regions, whereas comparisons covering a wide range of cloud conditions and meteorological situations are needed. More detailed comparisons of passive *N*_d_ retrievals with those using CALIOP (Hu et al., 2007; Zeng et al., 2014) may also be informative regarding *f*_ad_ since the latter retrieval does not rely on the assumption of an *f*_ad_ value. Polarimetry instruments may also be useful for characterizing *k* values directly from their retrievals.

Nevertheless, it was found that *N*_d_ uncertainty was dominated by errors in *r*_e_ for both the pixel level and 1° × 1° average, and so characterizing the other uncertainties will be unhelpful without also reducing *r*_e_ uncertainty. A first step would be to improve the characterization of *r*_e_ biases, which again would benefit greatly from more in situ comparisons. Detailed comparisons to polarimetry data (e.g., POLDER and future missions) would be beneficial since such instruments are potentially less biased. Questions regarding whether they are strongly affected by cloud top entrainment remain; however, aircraft observations are again likely to be useful here. It is also not clear how much the restriction of their retrievals to regions of low *r*_e_ variability limits their utility in examining *r*_e_ errors for passive instruments, since a characterization for heterogeneous clouds is also needed. Tests of retrievals on realistic known cloud fields (e.g., from LES models or reconstructed from retrievals) have also proven very useful; more such studies for a range of conditions are recommended, along with a better characterization of whether such models adequately capture the real‐world cloud variability that gives rise to *r*_e_ biases.

There now exist techniques to correct for the subpixel heterogeneity *r*_e_ bias based on 1‐D radiative transfer theory (Zhang et al., 2016). Werner et al. (2018) show that this works well for a limited number of stratocumulus cases, but further studies are needed to demonstrate how well this correction works in other situations and cloud types and whether instruments like MODIS make the needed measurements (e.g., a variability measure of the SWIR reflectances) at a high enough resolution. Very high resolution satellite data (as used in Werner et al., 2018) or that from airborne remote sensing will be useful for this but is currently under utilized. Given the ever increasing computation power available, retrievals that make use of 3‐D radiative transfer might be utilized to avoid the problematic plane parallel independent pixel approximation. It is also theoretically possible to avoid the use of the *r*_e_ retrieval altogether in *N*_d_ retrievals by making use of passive microwave remote sensing of cloud liquid water path (LWP). However, there are issues with the comparatively large footprint of microwave instruments and such approaches still require a *τ*_c_ retrieval. This approach can alternatively replace *τ*_c_ with LWP within *N*_d_ retrievals, which, while still using *r*_e_, may be useful for situations with aerosol layers above the cloud.

Cumulus clouds are more heterogenous and are likely to suffer larger *r*_e_ and hence *N*_d_ biases. Another caveat for such clouds is that the assumption of vertically uniform *N*_d_ may not hold, although a technique that may be able to deal with this has been described in this review (section 4.5). This uses high‐resolution (375 m) passive satellite observations of *r*_e_ to infer cloud base *N*_d_ (and potentially CCN) for deep convection. Further validation of such techniques would be very useful. This method may prove useful for trade cumulus clouds, but given their smaller size, this will likely necessitate even higher resolution retrievals. In situations where direct retrievals of *N*_d_ are difficult or impossible reanalysis‐based methods that combine satellite retrieval information with aerosol model data may prove useful (see section 4.6).

In this review we compared three climatological satellite *N*_d_ data sets: two from the MODIS instrument and one from AATSR using the ORAC retrieval. The two MODIS data sets (Bennartz & Rausch [2017, BR17] and Grosvenor & Wood [2014, GW14] agreed closely for the stratocumulus regions. For nonstratocumulus tropical and subtropical regions *N*_d_ from BR17 was less than that from GW14 by up to ∼50%. This comparison highlights that ad hoc assumptions applied to filter the sample based on cloud fraction, from a threshold of 70% in Painemal and Zuidema (2010), to 80% (GW14), to none at all (BR17), can have profound effects on the quantitative *N*_d_ estimate. Differences could be due to potential retrieval improvements but could also be caused by the observation that *N*_d_ and cloud fraction are typically positively correlated (Gryspeerdt et al., 2016). The seasonal cycle depictions (Figure 8) are also potentially affected. The requirement for the 3.7‐μm channel *r*_e_ to be larger than the 2.1‐μm channel value (and that larger than the 1.6‐μm retrieval) in BR17 is also likely to be a cause of discrepancies and sampling differences. Further work is needed to clarify how such choices affect *N*_d_ climatologies in order to ascertain which are optimal. At higher latitudes ( ≳50°N or S) *N*_d_ from BR17 was higher than that from GW14 by up to ∼30%. This was attributed to a lack of screening for high SZAs retrievals in BR17, which has been shown to cause *N*_d_ overestimates (see section 2.4.5). The AATSR‐ORAC *N*_d_ was 7% larger on average than that from GW14 but showed large overestimates of around 50% in the stratocumulus regions; this was attributed to a higher *τ*_c_ retrieval from AATSR‐ORAC. The average root mean square differences between the three data sets was around 20%, giving an estimate of uncertainty, albeit one based on instruments that use similar input data.

Issues regarding the seasonal cycle of *N*_d_ from these data sets were raised, for example, in the East China Sea where the *N*_d_ cycle is the opposite to that expected from the aerosol seasonal cycle. Possible reasons for this were discussed, which were related to the predominance of stratocumulus clouds in the winter and cumuliform clouds in the summer; this may lead to less accurate *N*_d_ retrievals in the summer or a physical effect from the concentration of pollutants in the boundary layer. More research is needed to determine whether this is the case and what the mechanisms are.

Following the issues raised in this review, some recommendations are made for the best practices for the adiabatic *N*_d_ retrieval from passive satellite instruments that is described in section 2:

1. Restrict pixel‐level *τ*_c_ to >5 since this greatly reduces the vertical penetration depth bias for *r*_e_ (section 2.3.1) and instrument uncertainty errors in *τ*_c_ and *r*_e_ (section 2.4.7).
2. Restrict the SZA to <65° and the VZA to <55° since large biases in *τ*_c_ and *r*_e_ retrievals have been demonstrated at larger angles (section 2.4.5).
3. Restrict retrievals to stratocumulus clouds since a number of the assumptions made for the retrieval break down for cumulus clouds with uncharacterized consequences for the uncertainty (section 2.3). Stratocumulus clouds are also more spatially homogenous, which reduces *r*_e_ biases.
4. Steps to limit retrievals to more homogeneous cloud fields should be considered even within stratocumulus. Methods to do this might include restricting to high cloud fractions as measured over large regions, for example, over 1° ×1° (see also section 3 and Wood & Hartmann , 2006); using the subpixel heterogeneity information available for the visible wavelength channels (section 2.4.1); or using the difference between *r*_e2.1_ and *r*_e3.7_ as a proxy for subpixel heterogeneity (section 3.2). However, the efficacy of these methods in terms of reducing the *r*_e_ bias has not been well characterized and it is recommended that further work is undertaken to do this.
5. Many studies suggest that the 3.7‐μm *r*_e_ retrieval is less prone to heterogeneity biases than the 2.1‐μm (or 1.6 μm) retrieval and so is likely the better choice for *N*_d_ retrievals, which are strongly affected by such biases. The 3.7‐um *r*_e_ is also less affected by the vertical penetration depth bias.

Approaches to better constrain *N*_d_ from ground‐based remote sensing now exist, with the advantage that they provide greater spatial and temporal detail compared to satellite observations. Basic methods combine radar and/or lidar with passive microwave observations of LWP to obtain *N*_d_, which is generally assumed to be vertically constant. Such techniques are more sensitive to assumptions made regarding the DSD than passive satellite, and also stringent requirements need to be placed upon the accuracy of the observations in order to reduce uncertainty to a level where they would serve as a useful references for the validation of space‐based *N*_d_ retrievals and climatologies. Some methods improve on this through the use of Doppler radar and cloud condensational growth models, allowing the profile of *N*_d_ to also be derived. More advanced methods combine information from different coincident measurements (radar, lidar, and shortwave spectral radiometers) using Ensemble Kalman Filter techniques that allow the inclusion of 3‐D scanning instruments and take 3‐D cloud radiative effects into account. The latter is likely particularly important for cumulus clouds. Another advantage is that no assumption for the liquid water content (*L*) profile is needed. Techniques using Raman lidar with two fields of view also allow the derivation of profiles of *N*_d_ along with *r*_e_, extinction coefficient (*β*_ext_), and *L* with reported uncertainties of 25–75%. Depolarization measurements using lidar also allow the determination of column mean *N*_d_ (with an uncertainty of ∼25–50%), *r*_e_ and *L* profiles and estimates of *f*_ad_. The latter is an input into the passive satellite retrieval of *N*_d_, and thus such measurements might prove useful for reducing uncertainty in the more traditional *N*_d_ retrievals.

The combination of ground‐based and satellite measurements of *N*_d_ and related cloud properties has been little utilized in the literature so far but might have the potential to enhance both types of retrievals and to characterize errors given the relative advantages of each. This is likely to be more useful for geostationary satellites that can view the region of the ground‐based instruments over long periods. Intercomparisons between data sets produced from satellite and ground‐based instruments may also give an estimate of the uncertainties. However, we caution that ground‐based methods also need further validation from in situ observations and that ground‐based retrievals of *N*_d_ are likely problematic in precipitating clouds.

In summary, there exist several ways to determine cloud droplet concentration via remote sensing, which is tantalizing given the large spatial and temporal coverage that this allows. Nevertheless, the uncertainties of these approaches are not well characterized, mainly due to the lack of validation studies covering different cloud regimes using in situ data, which, while not without its problems, is likely the most accurate method of determining *N*_d_ that we possess. Furthermore, in situ data are immensely useful in terms of characterizing the whole cloud profile, thus giving a detailed picture of the causes of biases. The problem is probably not a lack of data since numerous aircraft data sets exist but perhaps a lack of opportunity for such studies to be performed. From a satellite perspective, the use of active sensors, polarimetry, and high‐resolution instruments, along with bias correction procedures for more traditional methods, are particularly exciting recent developments. Likewise, new ground‐based techniques are providing fresh ways to study *N*_d_ at high resolution. Integrating several of these retrieval methods to study the same clouds would be likely to lead to new insights into the problem but is challenging and would require significant cooperation. There is also an urgent need for studies in cloud regimes that are more problematic for remote sensing, that is, outside of the stratocumulus regions.
